# Classification of Plant Endogenous States Using Machine Learning-Derived Agricultural Indices

**DOI:** 10.34133/plantphenomics.0060

**Published:** 2023-06-27

**Authors:** Sally Shuxian Koh, Kapil Dev, Javier Jingheng Tan, Valerie Xinhui Teo, Shuyan Zhang, Dinish U.S., Malini Olivo, Daisuke Urano

**Affiliations:** ^1^ Temasek Life Sciences Laboratory, National University of Singapore, Singapore, Singapore.; ^2^ Department of Biological Sciences, National University of Singapore, Singapore, Singapore.; ^3^ Translational Biophotonics Laboratory, Institute of Bioengineering and Bioimaging, Agency for Science, Technology and Research (A*STAR), Singapore, Singapore.; ^4^ Institute of Materials Research and Engineering (IMRE), Agency for Science, Technology and Research (A*STAR), Singapore, Singapore.

## Abstract

Leaf color patterns vary depending on leaf age, pathogen infection, and environmental and nutritional stresses; thus, they are widely used to diagnose plant health statuses in agricultural fields. The visible-near infrared-shortwave infrared (VIS-NIR-SWIR) sensor measures the leaf color pattern from a wide spectral range with high spectral resolution. However, spectral information has only been employed to understand general plant health statuses (e.g., vegetation index) or phytopigment contents, rather than pinpointing defects of specific metabolic or signaling pathways in plants. Here, we report feature engineering and machine learning methods that utilize VIS-NIR-SWIR leaf reflectance for robust plant health diagnostics, pinpointing physiological alterations associated with the stress hormone, abscisic acid (ABA). Leaf reflectance spectra of wild-type, *ABA2*-overexpression, and deficient plants were collected under watered and drought conditions. Drought- and ABA-associated normalized reflectance indices (NRIs) were screened from all possible pairs of wavelength bands. Drought associated NRIs showed only a partial overlap with those related to ABA deficiency, but more NRIs were associated with drought due to additional spectral changes within the NIR wavelength range. Interpretable support vector machine classifiers built with 20 NRIs predicted treatment or genotype groups with an accuracy greater than those with conventional vegetation indices. Major selected NRIs were independent from leaf water content and chlorophyll content, 2 well-characterized physiological changes under drought. The screening of NRIs, streamlined with the development of simple classifiers, serves as the most efficient means of detecting reflectance bands that are highly relevant to characteristics of interest.

## Introduction

Recent improvements in technology and artificial intelligence have enabled their gradual integration in agricultural practices. In precision and predictive agriculture, a broad array of environmental, horticultural, and plant physiological information are collected in real time and interpreted by machine learning (ML) models with the goal of improving agricultural output [1–3]. The means in which productivity is improved should be robust and efficient, to replace traditional methods of analyzing plant samples, which are destructive, time-consuming, and unsuitable for real-time data acquisition.

An understanding of plant stress responses is crucial to tackle the issue of improving agricultural outcomes. Phytohormones such as abscisic acid (ABA) often feature as a centrepiece in such studies due to its involvement in coordinating plant responses to environmental stresses [4,5]. ABA induces the closure of stomatal pores under drought, thereby preventing transpiration and water loss from leaves [6]. ABA is synthesized from β-carotene through a series of evolutionarily conserved catalytic reactions. The Arabidopsis ABA2 gene encodes a short-chain dehydrogenase/reductase that catalyzes the final step of ABA biosynthesis [7]. The ABA2-overexpressing mutants show delayed seed germination and higher tolerance to salinity stress compared to wild-type plants [8]. On the other hand, the null mutants for ABA2 (*aba2*) reduce endogenous ABA levels drastically [9,10] and have stomata that are perpetually open [9]. This results in rapid water loss in the *aba2* mutant compared to wild-type plants. ABA is also known to regulate metabolic pathways that increase amino acid and sugar contents [10,11]. Similar accumulations of primary metabolites occur in wild-type plants under drought, especially at earlier stages of drought stress [12–14]. Continuous, long-term treatment with ABA also reduces chlorophyll content and chloroplast division, leading to leaf yellowing [15]. By inducing both physiological and physical changes in leaves, ABA controls plant adaptation in response to drought and other stressors in the environment.

Improvements in spectroscopic techniques have enabled the real-time monitoring of plant health in a noninvasive manner [16]. Among them, visible (VIS, 400 to 700 nm), near infrared (NIR, 700 to 1,100 nm), and shortwave infrared (SWIR, 1,100 to 2,500 nm) reflectance spectroscopy (VIS-NIR-SWIR) measures the reflectance and/or transmittance of light by plants over a range of wavelengths. VIS-NIR-SWIR spectroscopy has been used to monitor the health status and marketability of crops [2,17]. Chlorophyll, anthocyanin, and water in leaves exhibit strong absorption peaks within the VIS-NIR-SWIR wavelengths, which provide essential information for plant health diagnostics. However, leaf reflectance also varies depending on the angle and surface structure of leaf [18], making data interpretation increasingly challenging and complex. To overcome these shortcomings, the ratio of 1 reflectance peak of interest to a reference peak, reflectanceratioindex=RefiRefj, was employed as general indicators of plant stress [19,20], and to estimate chlorophyll [21,22] and water levels [23–28]. Normalized reflectance index (NRI), $\mathrm{NRI}=\frac{\mathrm{ref}\left(i\right)-\mathrm{ref}\left(j\right)}{\mathrm{ref}\left(i\right)+\mathrm{ref}\left(j\right)}$, is another common strategy to normalize reflectance at 2 separate wavelengths [29,30]. Several NRIs using more than 2 wavelengths were also proposed for better estimation of phytopigments and nitrogen contents [26,31,32].

Despite the development of simple equations for various applications, spectroscopic technologies have not been widely employed in studying chronic physiological disorders, including chronic hormonal imbalance. Here, using ABA genetic mutants as a case study, we aim to construct highly interpretable and accurate support vector machine (SVM) classifiers using small numbers of spectral features obtained from the generation and screening of de novo NRIs. As the chronic stimulation by ABA is tightly associated with various stress responses, the dissection of hormone-dependent changes of leaf spectra would augment our understanding of the physio-spectral relationship of plant stress response. ML-guided selection of novel NRIs is likely more relevant compared to reflectance ratio indices and/or NRIs that may be nonspecific in nature.

## Materials and Methods

### Experimental and technical design

The overall workflow of our VIS-NIR-SWIR analysis consists of 4 steps: (1) pre-processing: smoothing and resampling of spectral data, (2) generation of de novo NRIs, (3) screening of customized nonredundant NRIs using analysis of variance (ANOVA) *F*-value scores and Pearson’s correlation coefficients (PCCs), and (4) construction of ML model and permutation test. As a model case, spectral changes related to drought and ABA responses were identified. The individual steps 1 to 4 are explained below in more detail.

### Plant preparation and drought treatment

ABA2 OE 4-4 (ABA2-ox, Columbia ecotype) [8] and *gin1-3* (*aba2*, Columbia ecotype) [7] seeds were provided by Cheng Wan-Hsing’s Lab. Col-0, ABA2-ox and *aba2* seeds were surface sterilized and germinated on Murashige and Skoog media with vitamins. Seeds were stratified for 3 d at 4 °C (with 1 μM gibberellic acid). After stratification, seeds were grown in a growth chamber under short-day conditions (22 °C, 8-h light/16-h dark cycle, 70 μmol m^−2^ s^−1^ light intensity and 70% relative humidity). After 7 d, Arabidopsis seedlings were transferred to soil and grown under the same short-day conditions.

Heirloom lettuce seeds (*Lactuca sativa var. Little Gem*) were surface-sterilized using a 10% bleach solution containing Triton X-100. Seeds were then germinated on Murashige and Skoog basal media with 1% agar in a plant chamber at 22 °C under 24-h continuous light and ~90 μmol m^−2^ s^−1^ light intensity. After 4 d, seedlings were transferred to soil and grown at 22 °C under a 16-h light/8-h dark cycle, ~150 μmol m^−2^ s^−1^ light intensity and 70% relative humidity.

Before drought treatment, all plants were watered to keep the soil wet. When the lettuce and Arabidopsis plants reached 25 d old and 49 d old, respectively, watering was withheld. At days 3 and 5 after the start of drought treatment, water was added to control plants only. Day 7 and 10 postdrought treatment readings were done for Arabidopsis only. VIS-NIR-SWIR spectroscopy and sample harvesting for biochemical measurements were taken in the afternoon 4 to 6 h after watering.

### VIS-NIR-SWIR spectroscopy

Leaf reflectance spectra from 350 to 2,600 nm were collected from the 2 biggest leaves (Arabidopsis) or third and fourth true leaves (lettuce) at 4 different positions per leaf using a VIS-NIR-SWIR fibered spectrometer (ARCoptix, Switzerland). The spectrometer was calibrated using a dark reading and white balance. The obtained spectra were resampled with a moving average over 4 nm in wavelength and then smoothed with a Savitzky–Golay filter with the window size of 11 and the polynomial order of 2. The spectral data were trimmed from 350–2,600 nm to 400–2,400 nm to exclude noisy edge wavelengths, the baseline (minimum value of each spectrum) was corrected to be 0.01. Conventional agricultural indices were obtained with a 4-nm window using the equations shown in Supplementary Materials.

### De novo screening of NRIs

De novo NRIs were calculated for all possible pairs of wavelengths using the following formula,

$$\text{Normalized}\;\text{Reflectance}\;\text{Index}=\frac{\mathrm{ref}\;i-\mathrm{ref}\;j}{\mathrm{ref}\;i+\mathrm{ref}\;j}$$

where ref *i* or *j* represents leaf reflectance at wavelength *i* or *j*. NRIs related to drought stress were selected by the *F* score feature selection algorithm (cutoff value = 20). The first 2 sets of NRIs were selected based on the differences between Col-0 and ABA2-ox or *aba2* mutants, while the third set was based on the differences between drought and watering condition in Col-0. The selected NRIs were ranked and selected based on the ANOVA *F* scores (greater than 20 for all Arabidopsis classification models), and redundant NRIs with PCC equal to or greater than 0.75 were excluded. These selected NRIs were used to build classification models.

### Classification model with a small number of spectral features

We constructed 3 models to classify (a) watering vs drought conditions in all 3 genotypes, (b) *aba2* from other 2 genotypes (Col-0 and *ABA2*-ox), and (c) *ABA2*-ox mutant from other 2 genotypes (Col-0 and *aba2*). The top-ranked NRIs (up to 20 NRIs) were selected with recursive feature elimination (RFE) with a random forest (RF) model with the number of estimators set at 100. The classifiers were built with SVM with radial basis function (RBF) kernel, RF and logistic regression (LogReg) models with the Sklearn python package. Using 85% of the top-ranked NRI datasets, hyperparameters (C for SVM, number of estimator and max depth for RF, and C for LogReg) were optimized through 50 iterations by the Optuna python package with *k*-fold cross validation with *k* = 5. The performance of the optimized models was evaluated with Repeats Stratified K-Fold cross validation (number of data split = 3, number of repeats = 10), as well as applying the finalized models on testing dataset (15% of NRI datasets).

### Partial least squares (PLS) regression

The PLS regression analysis was carried out using the PLSRegression function in the Scikit-learn (sklearn) library. Firstly, raw spectra were transformed to first derivative (D1) values and smoothed with a Savitzky–Golay filter with the window size of 11 and the polynomial order of 2. Using the D1 values, 2 PLS models were constructed predict relative chlorophyll content and leaf water content. The optimal combination of spectral bands and component numbers for each model was determined by selecting the point with the lowest mean squared error, which was calculated using the sklearn library. Additionally, a 10-fold cross-validation was performed using the cross_val_predict function from the Scikit-learn library to evaluate the predictive performance of the PLS models.

### Relative chlorophyll content measurements

The SPAD 502-Plus (Konica Minolta, Japan) was used to measure the relative chlorophyll content of Arabidopsis plants. SPAD readings were taken at 2 locations on the biggest 2 leaves of each plant. The positions chosen covered the areas used for VIS-NIR-SWIR spectroscopy. The mean of 4 readings from each plant was calculated and taken to be the average SPAD value for the whole plant.

### Leaf water content

A single leaf was harvested from each plant and the fresh weights for each sample were measured. Leaf samples were then dried in a 70 °C oven for 24 h. On the following day, dry weights of each leaf sample were measured. After deducting the dry weight from the fresh weight, the leaf water content of each leaf can be calculated as per the following formula:

$$\text{Leaf Water Content},\mathrm{LWC}\left(\%\right)=\frac{\text{Fresh}\;\text{Weight}\left(g\right)-\mathrm{Dry}\;\text{Weight}\left(g\right)}{\text{Fresh}\;\text{Weight}\left(g\right)}\times 100$$

### Biochemical analysis of anthocyanin content

Arabidopsis leaves harvested from control and drought treated plants were used for anthocyanin extraction. Firstly, the fresh weights of Arabidopsis leaves were measured. Samples were placed in microfuge tubes and snap-frozen in liquid nitrogen. The anthocyanin extraction was based on the procedure previously described [33]. In short, 300 μl of acidified methanol was added to ground leaf samples for overnight extraction. Water and chloroform were added to samples, followed by centrifugation of samples. The top phase was extracted and diluted with 60% acidified methanol.

Two replicates of 200 μl per sample were pipetted into a 96-well microplate. Total anthocyanin content (per gram fresh weight) was determined by measuring the absorbance at 530 nm (*A*_530_) and657 nm (*A*_657_) using a Spark multimode microplate reader (Tecan, Switzerland). Anthocyanin content was determined using the following formula [34]:

$$\begin{array}{l} \text{Relative}\;\text{units}\;\text{of}\;\text{anthocyanins}/g\;\text{fresh}\;\text{weight}\;\text{of}\;\text{tissue}\\ ={\mathrm{OD}}_{530}-\left(0.25\times {\mathrm{OD}}_{657}\right)\times \text{extraction}\;\text{volume}\left(\mathrm{mL}\right)\times \frac{1}{\text{fresh}\;\text{weight}\left(g\right)} \end{array}$$

### Biochemical analysis of carotenoid content

Carotenoids were extracted as previously described [35]. Approximately 100-mg fresh weight of Arabidopsis leaves was harvested and snap-frozen in liquid nitrogen. After grinding samples, a 4:3 ratio of ethanol to hexane (containing 1% butylated hydroxytoluene) was added to each sample. All samples were vortexed for 30 s at 22 °C. Samples were then sonicated for 5 min. Following sonication, samples were centrifuged at 2,500 x g, 4 °C for 5 min. The top phase was transferred to a new tube and dried using a nitrogen stream. Samples were then redissolved in 1 ml of chloroform containing 1% butylated hydroxytoluene.

Two replicates of 200 μl per sample were pipetted into a 96-well microplate. Carotenoid content was determined by measuring absorbance at 480, 648, and 666 nm (*A*_480_, *A*_648_, and *A*_666_, respectively) using a Spark multimode microplate reader (Tecan, Switzerland). Carotenoids (C_x+c_), chlorophyll a (C_a_), and chlorophyll b (C_b_) content was determined using the following formulae [36]:

$$\begin{array}{c} C_{a}=10.91A_{666}-1.2A_{648}\\ C_{b}=16.38A_{648}-4.57A_{666}\\ C_{x+c}=\left(1000A_{480}-1.42C_{a}-46.09C_{b}\right)/202 \end{array}$$

## Results

### Distinct leaf reflectance signatures of *ABA2* overexpressing and deficient mutants under drought stress

**Fig. 1.**
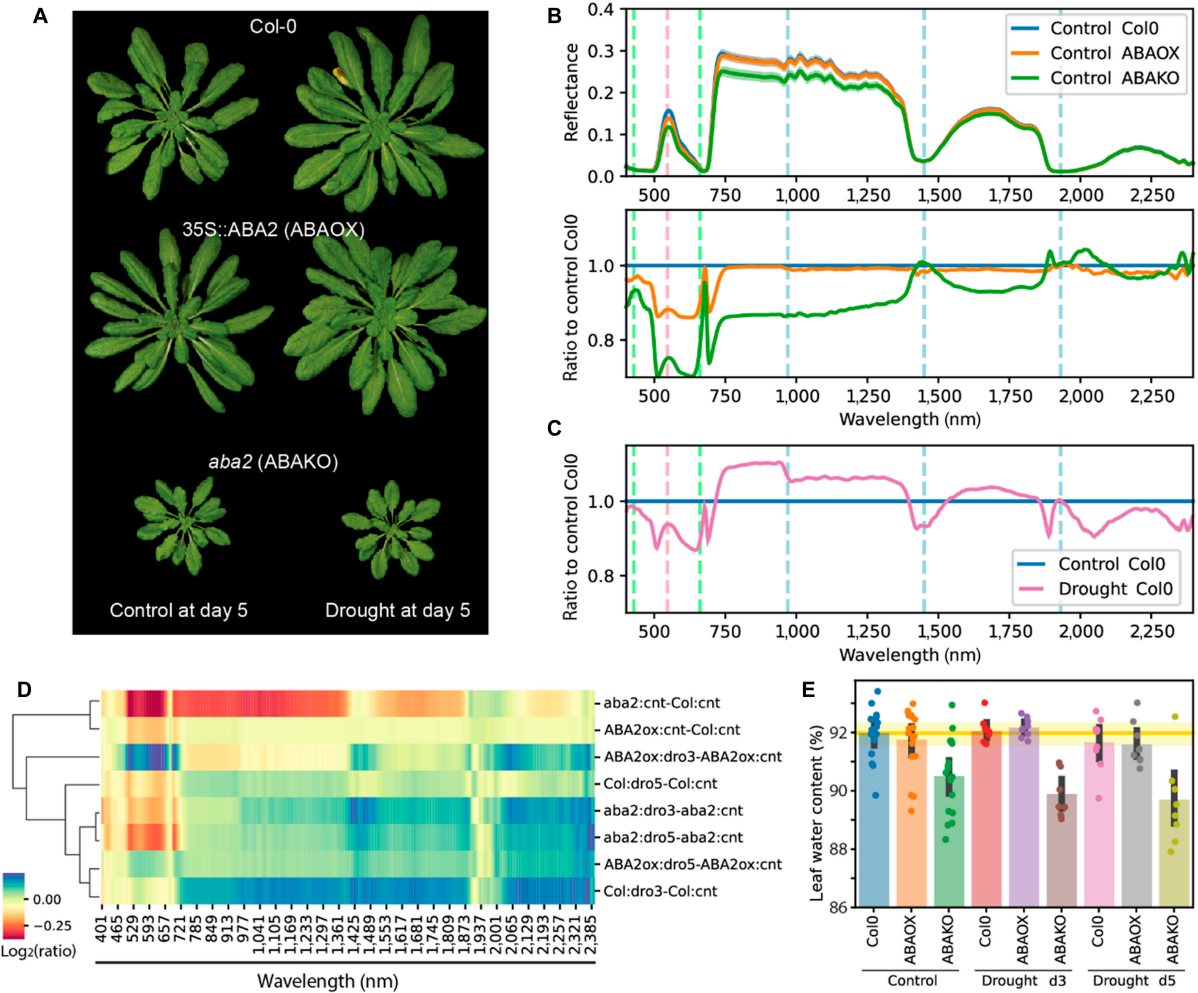
Leaf reflectance spectra of Col-0, *ABA2-ox*, and *aba2* plants under watering and drought conditions. (A) Representative images of Arabidopsis wild type and mutants on the fifth day under watering and drought conditions. (B) Leaf reflectance spectra from Col-0, *aba2*, and ABA2-ox plants under watering condition. Solid line and transparent band represent the mean value and 95% confidence interval, respectively. The bottom graph shows reflectance ratio of mutants to Col-0. (C) Reflectance ratio of drought to watering condition at days 3 and 5 in Col-0. In (B) and (C), dashed vertical lines represent absorption spectra for chlorophyll (428 and 660 nm, green), anthocyanin (546 nm, red), and water (970, 1,450, and 1,930 nm, blue). (D) Heatmap showing reflectance ratio of drought (dro) to control (cnt) in individual genotype, or ratio of mutant (ABA2ox or aba2) to wild type (Col) under drought and control conditions. Heatmap colors represent the log_2_-transformed values of reflectance ratio. (E) Leaf water content in Arabidopsis leaves under well-watered and drought conditions (*n* = 3 per condition, for a total of 3 independent rounds of experiments). Individual points show raw data, bars show mean, while error bars show SD. The horizontal yellow line shows the mean value, while the yellow band shows 95% confidence interval of Col-0.

Leaf reflectance information over wavelengths 350 to 2,600 nm was recorded from 7- to 8-week-old wild type (Col-0), the ABA-deficient mutant (*aba2*), and the *ABA2*-overexpressing line (ABA2-ox) at days 3, 5, 7, and 10 (Fig. 1 and Figs. S1 to S3) of drought treatment. Raw leaf reflectance data with 1-nm spectral resolution was first smoothed using a Savitzky–Golay filter. Wavelengths shorter than 400 nm and longer than 2,400 nm were excluded due to noisy reflectance signatures. Outliers in the data collected were further removed using an Isolation Forest algorithm (Fig. S1), yielding a total of 1,277 leaf reflectance data for downstream analysis.

Under normal growth conditions in soil, the *aba2* plants were smaller in size relative to Col-0, but there were no noticeable differences in leaf color (Fig. 1A and Fig. S2). The ABA2-ox line had minimal differences in leaf morphology and color compared to Col-0. From days 3 to 10 of drought treatment, there were no visible differences between control and drought conditions in all genotypes (Fig. S2). Despite the unnoticeable changes, *aba2* plants showed distinct spectral signatures compared to Col-0 (Fig. 1B and Fig. S3). ABA2-ox plants had slightly decreased reflectance from 400 to 750 nm and had similar reflectance patterns from 750 to 2,400 nm compared to Col-0 under control conditions. The trend was consistent from days 3 to 10 of drought treatment (Fig. S3). *Aba2* plants under both control and drought conditions showed greatly reduced reflectance from 490 to 680 nm, followed by a moderate reduction in reflectance from 750 to 2,400 nm. The spectral change in *aba2* mutant was aggravated by drought treatment, especially at days 7 and 10 (Fig. S3C and D). A similar reduction in reflectance from 400 to 750 nm was observed in Col-0 plants under drought, but drought-stressed Col-0 plants had slightly increased reflectance compared to control condition from 750 to 2,400 nm (Fig. 1B and C).

Figure 1D shows the log_2_-transformed ratio of leaf reflectance spectra between drought and control conditions or between mutants and Col-0 plants. Similar to the spectral differences between *aba2* and Col-0 under control conditions (Fig. 1B), leaf reflectance from 490 to 680 nm was reduced in Col-0 and *aba2* plants under drought treatment, with a greater reduction at day 5. Minimal differences were detected at 2 water absorption peaks (1,450 and 1,930 nm) among any comparisons, despite the apparent reduction in leaf water content in *aba2* plants (Fig. 1E). This indicates that water loss in leaves, an indicator of drought stress severity, is uncorrelated with major spectral changes at earlier stages of drought stress. Hierarchical clustering analysis using cosine distances placed the spectral ratios between Col-0 and mutant plants under control condition (top 2 rows, Fig. 1D) distant from the comparisons between drought and control conditions. Taken together, the spectral comparisons revealed the decreased leaf reflectance at 490 to 680 nm is an early indicator of drought stress that is independent of water loss in leaves but could be related to endogenous ABA levels.

### De novo screening of NRIs, feature selection and classification

**Fig. 2.**
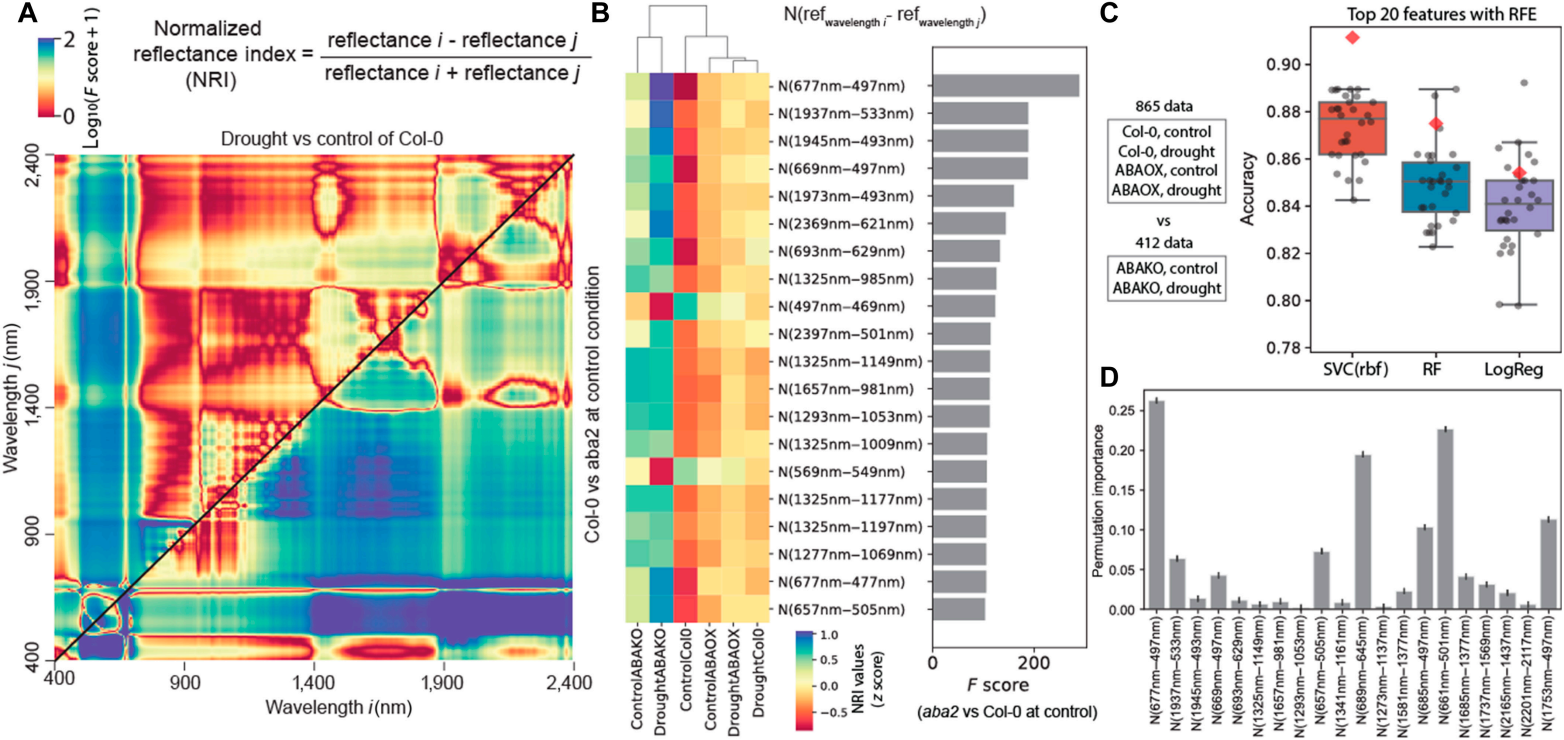
De novo screening of drought- and *aba2*-oriented NRIs. (A) All possible pairs of NRIs for a binary classification. Heatmap shows ANOVA *F* values for classification models; the comparison of NRIs in *aba2* mutant to those in Col-0 (lower right triangle) and the drought to watering condition in Col-0 (upper left triangle). (B) Nonredundant NRIs selected based on the *aba2* vs Col-0 comparison using ANOVA *F*-value scores and PCC. Top 20 NRIs were further selected with the RFE method. Heatmap shows *z*-score-normalized indices across 6 tested groups (3 genotypes × 2 conditions). The bar graph on the right shows *F* scores. (C and D) Classification models to separate *aba2* mutant from other 2 genotypes with the selected NRIs. Classifiers were built with SVM, RF, and LogReg algorithms with top 20 NRIs. Boxplots show classification accuracy with the training set by the Repeats Stratified K-Fold cross validation (*n* = 30, black dots). Red diamond shows the accuracy with the testing set. The SVM model exhibited higher accuracy compared to the RF and LogReg models, and its permutation importance is presented in (D). Bar graph in (D) shows the mean ± SD derived from 25 permutation tests.

Identifying NRIs relevant to the traits of interest provides a turnkey solution to monitor plant stress status. To identify optimal NRIs associated with drought and/or endogenous ABA level, we performed de novo screening of NRIs highly relevant to the comparisons of control vs drought conditions (Fig. 2A) and of Col-0 vs mutant plants (Fig. S4). In each wavelength pair (wavelengths *i* and *j*), NRIs were calculated using a formula of $\mathrm{NRI}=\frac{\mathrm{ref}\left(i\right)-\mathrm{ref}\left(j\right)}{\mathrm{ref}\left(i\right)+\mathrm{ref}\left(j\right)}$. The 2 sets of comparisons, *aba2* vs Col-0 under control and control vs drought in Col-0, showed similar patterns where the highest *F* score values were consistently observed with the NRIs containing wavelengths from 490 to 690 nm (Fig. 2A). The comparison of drought vs control in *aba2* also showed similar patterns of *F* scores but to a greater extent compared to those in Col-0 (Fig. S4A and B), while drought vs control in ABA2-ox revealed nearly zero *F* scores throughout all NRIs (Fig. S4C). These results suggest that the endogenous level of ABA has a substantial role in spectral changes caused by drought.

NRIs that were related to the comparison being made were kept and then ranked according to the samples’ *F* values (the degree of the variation between sample means over the variation within samples). NRIs that had low ANOVA *F* value (*F* ≤ 20) and high collinearity (|PCC| ≥ 0.75) were excluded. *Z*-scored, log_2_-transformed NRIs were plotted as a heatmap, and the *F* values were plotted for the top 20 NRIs selected with the RFE method (Fig. 2B and Figs. S5 and S6).

Clustering analysis with the top 20 NRIs of *aba2* vs Col-0 showed a clear distinction of *aba2* plants from ABA2-ox and Col-0, which had similar spectral profiles to each other. ABA2-ox data collected under both control and drought conditions were clustered closely to Col-0 under drought, implying that elevated ABA level partially mimics informative spectral changes caused by drought. Next, using the small, nonredundant set of NRIs, interpretable classifiers to distinguish *aba2* from other 2 genotypes (Col-0 and ABA2-ox) were built with SVM, RF, and LogReg models (Fig. 2C). The SVM classifier with the RBF exhibited the highest performance with the mean accuracy of 87.3% ± 1.4%. Permutation importance test showed only a small reduction of classification accuracy by shuffling each feature value randomly (Fig. 2D), suggesting the robustness of SVM classifier.

Similar interpretable classifiers were built to distinguish control vs drought in all 3 genotypes (Fig. S5), and ABA2-ox vs other 2 genotypes (Col-0 and *aba2*) (Fig. S6). The drought vs control comparison in Col-0 identified 72 NRIs that have low collinearity and high *F* value (|PCC| < 0.75, *F* > 20, Fig. 2A and Fig. S4A), among which the top 20 were further selected with the RFE method (Fig. S5A). A dendrogram generated with the top 20 NRIs was separated based primarily on the control vs drought differences rather than the genetic effects of ABA overexpression or deficiency (cluster heatmap, Fig. S5A). ML models built with the 20 NRIs classified control and drought groups regardless of genetic background, where the SVM classifier with RBF kernel showed highest mean accuracy of 80.4 ± 1.8% (Fig. S5B and C). The performance of these ML models was generally higher than those built with conventional agricultural indices as features (Fig. S7). As ABA2-ox plants exhibited minimal spectral changes from Col-0 (Fig. 1), we were able to identify only 9 nonredundant NRIs associated with the ABA2-ox vs Col-0 comparison (Fig. S6A), which resulted in the lower performance of classifiers (the highest accuracy of 66.0 ± 0.3% with SVM model, Fig. S6B and C). Taken together, the comparisons made between genotype and treatment groups reveal NRIs that are associated with drought stress and endogenous levels of ABA.

### Association of the selected NRIs with water and relative chlorophyll contents

**Fig. 3.**
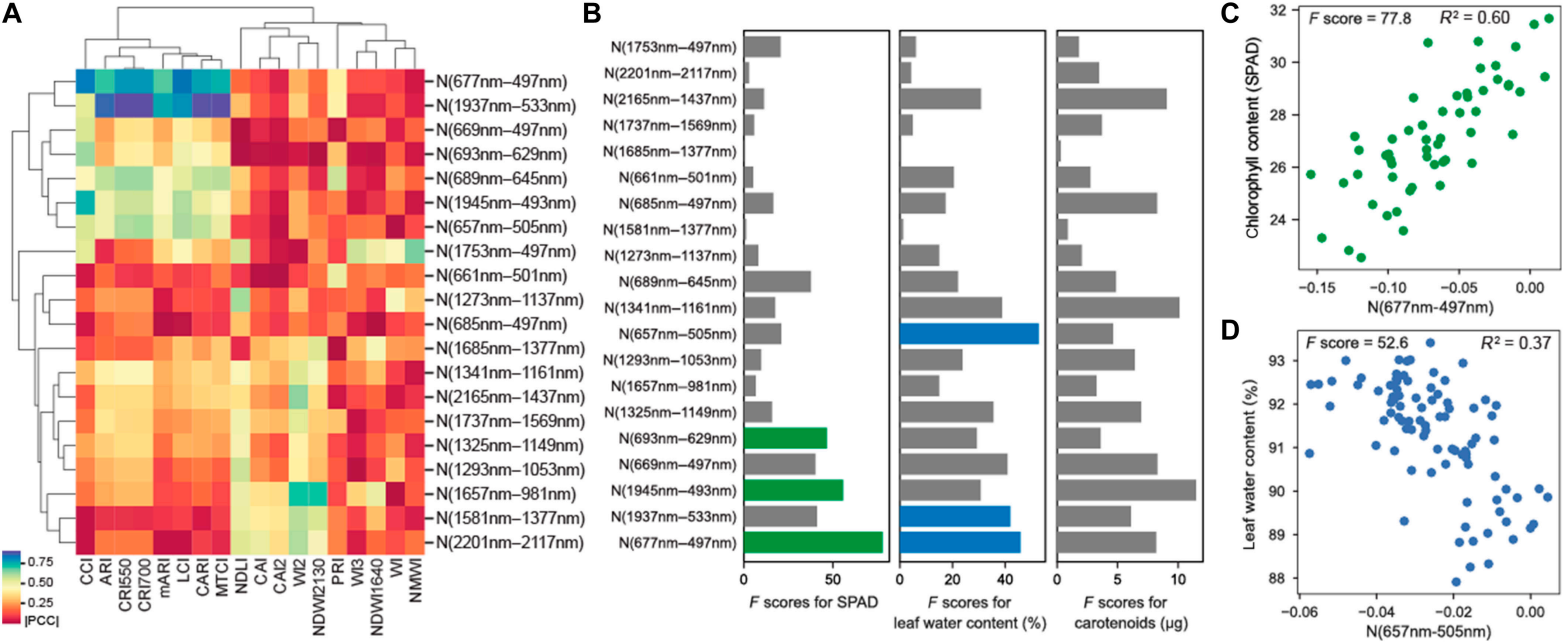
Physiological changes associated with *aba2*-oriented NRIs. (A) Correlation of *aba2*-oriented NRIs with conventional agricultural indices. The names of individual indices and equations are summarized in Supplementary Information. Colors in heatmap represent the absolute value of correlation coefficients (|R|). NRIs highlighted in orange or blue show high correlation with carotenoid/anthocyanin- or water-related agricultural indices, respectively. (B) Horizontal bar graphs show the feature importance (*F* score) correlated to experimentally determined relative chlorophyll content (*n* = 54), water content (*n* = 90), and carotenoid content (*n* = 54). The raw experimental data are presented in Fig. 1E and Fig. S8. Green and blue colors indicate top 3 NRIs associated with relative chlorophyll and water contents. (C and D) Scatterplot of *aba2*-oriented NRI values with relative chlorophyll content (C) and leaf water content measurements (D). The most important indices for chlorophyll (N[677 nm–497 nm]) and water (N[657 nm–505 nm]) were selected from (B). Top 5 indices based on F scores for chlorophyll, water, and carotenoid content are shown in Figs. S9 (relative chlorophyll), S10 (water), and S11 (carotenoids).

To check the possible overlap of the spectral features we picked up during the de novo NRI search, the correlation of these NRIs was checked against conventional agricultural indices (Table S1). A few NRIs screened from Col-0 vs *aba2* comparison showed a high correlation (|PCC| ≥ 0.75) to conventional agricultural indices, most among which were related to phytopigments and water contents such as leaf chlorophyll index, carotenoid reflectance index 550, and normalized differential water index with 2,310 nm (Fig. 3A). The plausible association of the selected NRIs with phytopigments and water contents were further evaluated by pairwise comparisons to biochemical results (Fig. 3B and Figs. S9 to S11). The ANOVA *F* values revealed that only small numbers of the selected NRIs (e.g., NRI(677 nm–497 nm)) were related to relative chlorophyll content (green bars, Fig. 3B) and leaf water content (blue bars, Fig. 3B), while the majority was unrelated to these 2 biochemical results as well as carotenoid content (gray bars, Fig. 3B). The top scored NRIs, NRI(677 nm–497 nm) and NRI(657 nm–505 nm), showed linear correlations to relative chlorophyll content and water content (*F* score = 77.8 and 52.6 respectively, Fig. 3C and D), albeit the correlation was generally low (Figs. S9 to S11).

PLS regression method was also applied to predict water content and relative chlorophyll content from original spectral data. Wavelengths associated with the 2 contents were widely distributed throughout the tested wavelength range; however, visible wavelengths from 450 to 750 nm and wavelengths near 2 water absorption peaks (1,450 and 1,900 nm) were mostly eliminated from the regression models (Fig. S12). The wavelengths selected through PLS regression were inconsistent with the top selected NRIs (Fig. 2) based on the comparisons of *aba2* vs Col-0 and control vs drought, further supporting the involvements of uncharacterized physiological changes in addition to the well-documented changes in water contents by drought stress. These results collectively suggest that water content and chlorophyll content partially explain the spectral changes observed during early drought response and/or changes in endogenous ABA levels.

### De novo screening for drought associated NRIs in lettuce

**Fig. 4.**
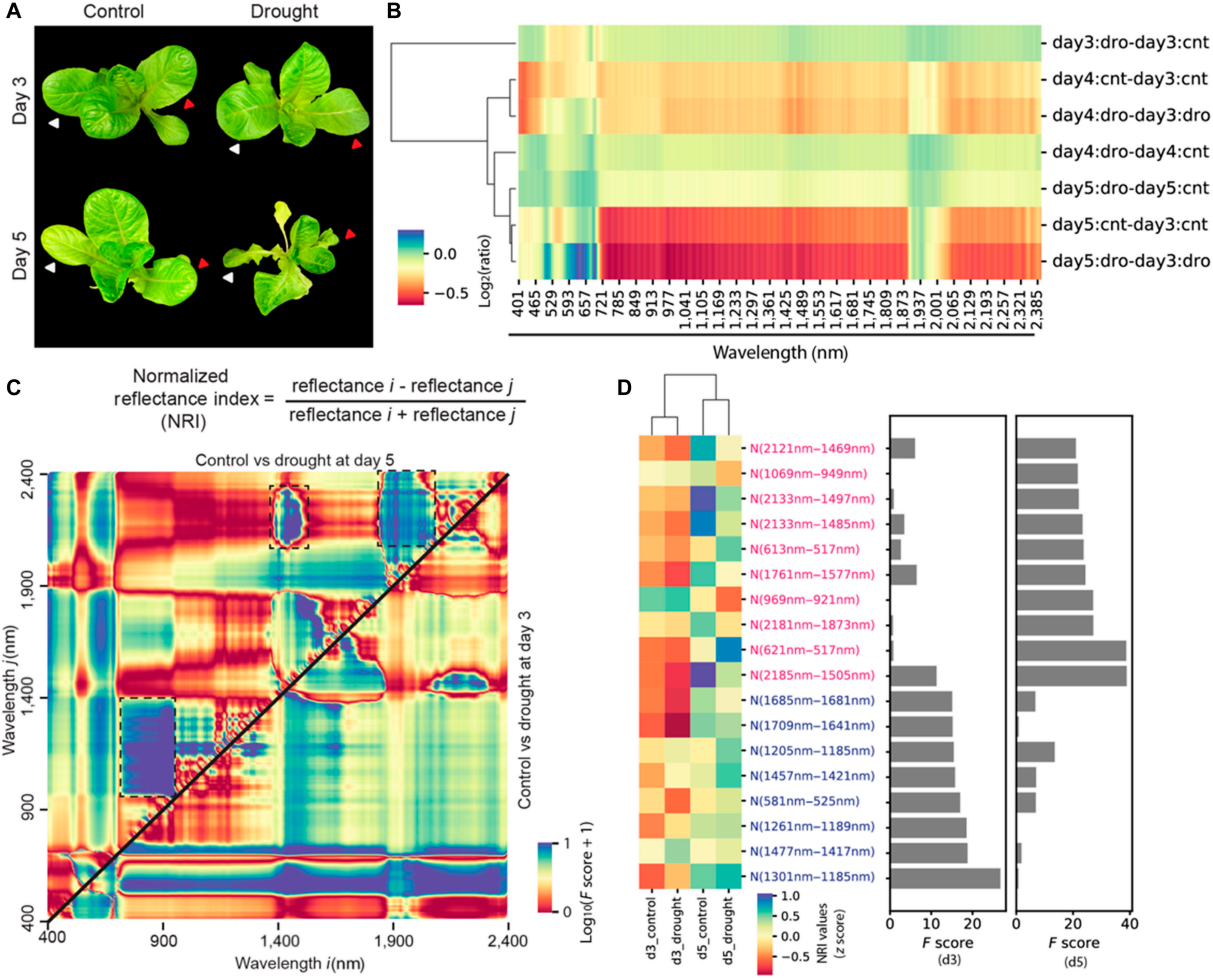
Drought-oriented NRIs in lettuce. (A) Representative images of lettuce under watering and drought conditions at days 3 and 5. (B) Reflectance ratio of drought (dro) to control (cnt) after different durations of drought treatment (days 3, 4, and 5 after watering was withheld). Heatmap also shows reflectance ratio to drought or watering condition at day 3. Colors represent the log_2_-transformed values of reflectance ratio. (C) De novo NRIs related to watering conditions in lettuce. Heatmap shows the log_10_-transformed *F*-value scores of the drought to watering condition at day 3 (lower right triangle) and day 5 (upper left triangle). (D) Selection of de novo agricultural indices based on *F*-value scores. Features with high collinearity (|PCC| ≥ 0.75) were excluded. Heatmap shows *z*-score-normalized indices across 4 tested groups. Blue and pink texts show reflectance indices from the drought vs control comparisons at day 3 and day 5 respectively. The bar graph on the right shows *F* scores.

We established a framework to screen reflectance spectra and NRIs that could be associated to drought stress and endogenous ABA level in Arabidopsis (Figs. 1 to 3). The screening strategy should be applicable to any species and stress types. To support this further, dwarf Romaine lettuce, *L. sativa*, was grown and subject to drought treatment, with data collection 3 to 5 d postdrought (Fig. 4A and Fig. S13). Similar to observations in Arabidopsis (Fig. 1B and C), leaf reflectance spectra at 490 to 680 nm and 750 to 2,400 nm decreased after 3 d of drought treatment (Fig. 4B). At days 4 and 5, drought caused the reduction in NIR and SWIR reflectance (Fig. 4B), while the difference at visible light wavelengths became marginal between drought and control samples. The marginal difference can be explained by the age-dependent yellowing of lettuce leaves even in the control group (Fig. 4A and Fig. S13F).

We next generated drought-related NRIs from the comparison of control vs drought at days 3 and 5 (Fig. 4C). The day 3 comparison showed high *F* score values from NRIs using the wavelengths of 490 and 680 nm. On the other hand, the day 5 comparison highlighted the importance of NIR wavelengths (dashed line boxes, Fig. 4C), suggesting that drought-associated changes in leaf reflectance could vary due to the severity and duration of drought as well as leaf age. Furthermore, similar to Arabidopsis (Figs. 1 to 3), a nonredundant set of NRIs (|PCC| < 0.75) was selected from the drought vs control comparison in lettuce (Fig. 4D). The top selected NRIs from the control vs drought comparison at day 5 (labeled in pink) contained pairs of wavelengths (NRI(613 nm–517 nm) and NRI(1,069 nm–949 nm)) that are similarly observed in Arabidopsis control and drought comparison (Fig. S14). On the other hand, 2 of the top selected NRIs at day 3 (NRI(1,301 nm–1,185 nm) and NRI(1,261 nm–1,189 nm)) overlapped with those found in Col-0 vs *aba2* under watering condition (Fig. S14), possibly implying the involvement of ABA into the early drought-related spectral changes in lettuce.

## Discussion

Leaf reflectance analyses with handheld devices and hyperspectral imaging provide quantitative information on plant physiological conditions. In addition, leaf reflectance varies depending on various physiological (e.g., leaf age and genetic backgrounds) and external factors (e.g., lighting condition, leaf thickness, and sensor sensitivity). NRIs are widely adopted as a means to reduce external effects related to different lighting conditions, leading to the improved predictive capability of chlorophyll, water, anthocyanins, and carotenoids content in plants [23,25–28]. Our study shows a novel method of building predictive models optimized for specific needs (ABA status and drought), combined with de novo NRI screening and ML techniques.

In this study, Arabidopsis genetic mutants, ABA2-ox and *aba2* plants, were adopted as tools to effectively identify normalized leaf reflectance indices that are associated with physiological statuses of plants (Figs. 1 to 3). With existing knowledge of genetic causes of abiotic stress, one can pinpoint Arabidopsis mutants resembling the phenotypic changes of common abiotic stress conditions that crops of interest commonly face. Thus, choosing suitable Arabidopsis mutants is essential for the development of NRIs and the downstream ML pipeline, which detects reflectance bands that are highly relevant to specific characteristics of interest. The spectral changes observed could be related to relative chlorophyll, water, carotenoids, and anthocyanin content. Using the band selection strategy from mutant leaf spectra could offer better precision of plant diagnostics (Fig. 2C), compared to existing agricultural indices that may be nonspecific in nature (Fig. 3A and Fig. S15).

The *aba2* plants invariably open stomata as a surrogate for drought stress and alter amino acid and sugar accumulations under drought. These physiological and morphological changes caused by the absence of ABA overlap with the early symptoms under drought stress. Consistently, we found that leaf reflectance collected from wild-type and mutant plants under control and drought conditions demonstrated the resemblance of the *aba2* mutant to drought stress in wild-type plants (Fig. 1B and C). However, little consistency between *aba2* mutation and early drought response at day 3 was observed for experimentally determined relative chlorophyll, carotenoid, anthocyanin, and water contents (Fig. S8). In addition, the spectral bands remained unchanged in ABA2-ox plants under drought at both days 3 and 5, consistent with previous reports on the drought tolerance phenotypes of ABA2-ox plants [8]. Together, these suggest that the comparable spectral changes caused by drought and ABA could not be attributed to the concentration of single pigments or leaf water contents rather to the combined effects of these and other physiological changes.

The comparison between the *aba2* mutant and Col-0 yielded the highest-scoring NRIs that could potentially reflect ABA-specific changes in leaf reflectance. Specifically, wavelengths between 493 and 533 nm were present in all of the top 5 highest scoring NRIs in the *aba2* vs Col-0 comparison (Fig. 2B). These wavelengths were also found in the top 20 highest scoring NRIs useful for distinguishing control vs drought conditions (Fig. S5A), but lower frequently and only ranking ninth or lower. Thus, the specific wavelengths used in the NRIs for the *aba2* Col-0 model could be ABA-specific, and their presence in the drought model could be linked to the known involvement of ABA in drought stress. Although some of the observed changes in spectra may not be the direct result of the absence of ABA, there exists an interesting correlation between genetic mutations and how they can aid our understanding of abiotic stress response. Further dissection of the comparable spectral changes will be achieved with a deeper understanding of the Arabidopsis mutants and how they are related to drought stress.

We employed feature engineering and selection strategies based on the design principle of NRIs, selecting one wavelength highly correlated with the traits of interest and another one that is independent or inversely correlated with the same trait. This method effectively decreases the high variation of raw reflectance data caused by leaf thickness, leaf microstructure, and lighting condition. Screening of de novo NRIs revealed various undocumented NRIs associated with ABA and drought stress, but mostly independent from conventional agricultural indices (Fig. 3A). Apart from chlorophyll and water content, numerous physical and chemical parameters can affect light absorption and scattering in leaves, including the thickness, trichomes, surface cuticle layer, internal cellular organization, physical damage to leaves, and flavonoids and phenolic compounds. Thus, further investigations of NRIs, coupled with biochemical quantification, are essential to enhance our understanding of the physical, chemical, and spectral properties of leaves. Nevertheless, some NRIs from the de novo screening exhibited moderate correlation with experimentally determined water and relative chlorophyll contents (Fig. 3B), suggesting that water and chlorophyll still constitute as the molecular signatures for plant health diagnostics based on leaf reflectance. In addition, we recognize that the high dimensionality of NRIs arising from band-by-band calculation potentially results in the corresponding bands of specific NRIs between different rounds of calculation. Nevertheless, these experimentally explainable and unexplainable NRIs used in tandem would provide more robust diagnostics of plant stresses compared to relying solely on existing agricultural indices.

In this work, Arabidopsis mutants are adopted to identify spectral changes associated with the biosynthesis pathway of ABA. A plethora of Arabidopsis genetic mutants deficient in specific metabolic pathways, signaling pathways, and small-molecule transporters have been generated and characterized physiologically. A potential expansion of this work will involve the collection and databasing of leaf reflectance information from these genetic resources. Through the scrutiny of leaf reflectance data, we will be able to understand spectral changes that pinpoint the genetic deficiencies present in biological pathways. In the present study, our data suggests the usefulness of using a genetic mutant in place of conventional treatment methods in identifying spectral variations related to hormone-dependent physiological changes. In the future, an extensive collection of leaf reflectance information could potentially aid the designing and optimization of robust NRIs and ML models that can ultimately be deployed for field use. Given that the *aba2* mutant exhibited spectral changes that are indicative of drought stress at an early stage (Figs. 1 to 3), the use of Arabidopsis mutants could also reduce the time required in plant stress experiments, increasing efficiency in the process.

## Data Availability

All authors confirm that all raw experimental data are available upon request.
