# Supplementary material for: Classification of Plant Endogenous States Using Machine Learning-Derived Agricultural Indices
Source: Plant Phenomics. 2023 Jun 27;5:0060. doi: 10.34133/plantphenomics.0060 (PMC10298216; doi:10.34133/plantphenomics.0060)
Supplement: Supplementary file 1 — Table S1 Figs. S1 to S15 [file plantphenomics.0060.f1.docx]

Supplementary Materials for

Classification of plant endogenous states using machine learning-derived agricultural indices

**Authors**

Sally Shuxian Koh^1,4*^, Kapil Dev^2^, Javier Jingheng Tan^1^, Valerie Xinhui Teo^3^, Shuyan Zhang^3^, Dinish U.S.^3*^, Malini Olivo^3*^, and Daisuke Urano^1,4*^

**Affiliations**

^1^Temasek Life Sciences Laboratory, National University of Singapore, Singapore.

^2^Translational Biophotonics Laboratory, Institute of Bioengineering and Bioimaging, Agency for Science, Technology and Research (A*STAR), Singapore.

^3^Institute of Materials Research and Engineering (IMRE), Agency for Science, Technology and Research (A*STAR), 2 Fusionopolis Way, Innovis #08-03, 138634, Singapore.

^4^Department of Biological Sciences, National University of Singapore, Singapore.

*Address correspondence to: [sally@tll.org.sg](mailto:sally@tll.org.sg), [dinish@imre.a-star.edu.sg](mailto:dinish@imre.a-star.edu.sg), [malini_olivo@imre.a-star.edu.sg](mailto:malini_olivo@imre.a-star.edu.sg), [daisuke@tll.org.sg](mailto:daisuke@tll.org.sg)

**This file contains**

Table S1

Figures S1 to S15

**Table S1.** Conventional Agricultural Indices

| **Agricultural Index** | **Equation** |
| --- | --- |
| Anthocyanin Reflectance Index (1) | $ARI=\frac{1}{ref550}-\frac{1}{ref700}$ |
| Modified Anthocyanin Reflectance Index (2) | $mARI=\left( \frac{1}{ref550}-\frac{1}{ref700} \right)\times ref1100$ |
| Carotenoid reflectance index at 550 nm (3) | $CRI550=\frac{1}{ref510}-\frac{1}{ref550}$ |
| Carotenoid reflectance index at 700 nm (3) | $CRI700=\frac{1}{ref510}-\frac{1}{ref700}$ |
| new Carotenoid Index (4) | $nCI=\frac{ref720}{ref521}-1$ |
| Photochemical Reflectance Index (5) | $PRI=\frac{ref531- ref570}{ref531 + ref570}$ |
| Chlorophyll / Carotenoid Index (6) | $CCI=\frac{ref532- ref650}{ref532 + ref650}$ |
| Leaf Chlorophyll Index (7) | $LCI=\frac{ref850- ref710}{ref850 + ref680}$ |
| MERIS Terrestrial chlorophyll index (8) | $MTCI=\frac{ref754- ref709}{ref709- ref681}$ |
| Modified Chlorophyll Absorption Ratio Index 710 (9) | $MCARI710=\left( (ref750-ref710 \right)-0.2\times(ref750-ref550))\times\frac{ref750}{ref710}$ |
| Water Ratio Index (10) | $WI=\frac{ref900}{ref970}$ |
| Water Ratio Index 2 (11) | $WI2=\frac{ref1300}{ref1450}$ |
| Water Ratio Index 3 (12) | $WI3=\frac{ref1350}{ref870}$ |
| Normalized Difference Water Index at 1640 nm (13) | $NDWI1640=\frac{ref858- ref1640}{ref858+ ref1640}$ |
| Normalized Difference Water Index at 2130 nm (13) | $NDWI2130=\frac{ref858- ref2130}{ref858+ ref2130}$ |
| Cellulose Absorption Index (14) | $CAI = 0.5 \times\left( ref2030+ref2210 \right)-ref2100$ |
| Cellulose Absorption Index 2 (15) | $CAI2 = 0.5 \times\left( ref2020+ref2220 \right)-ref2100$ |
| Normalized Difference Lignin Index (16) | $NDLI=\frac{\log\left( 1/ref1754 \right)- log(1/ref1680)}{\log\left( 1/ref1754 \right) + log(1/ref1680)}$ |

**
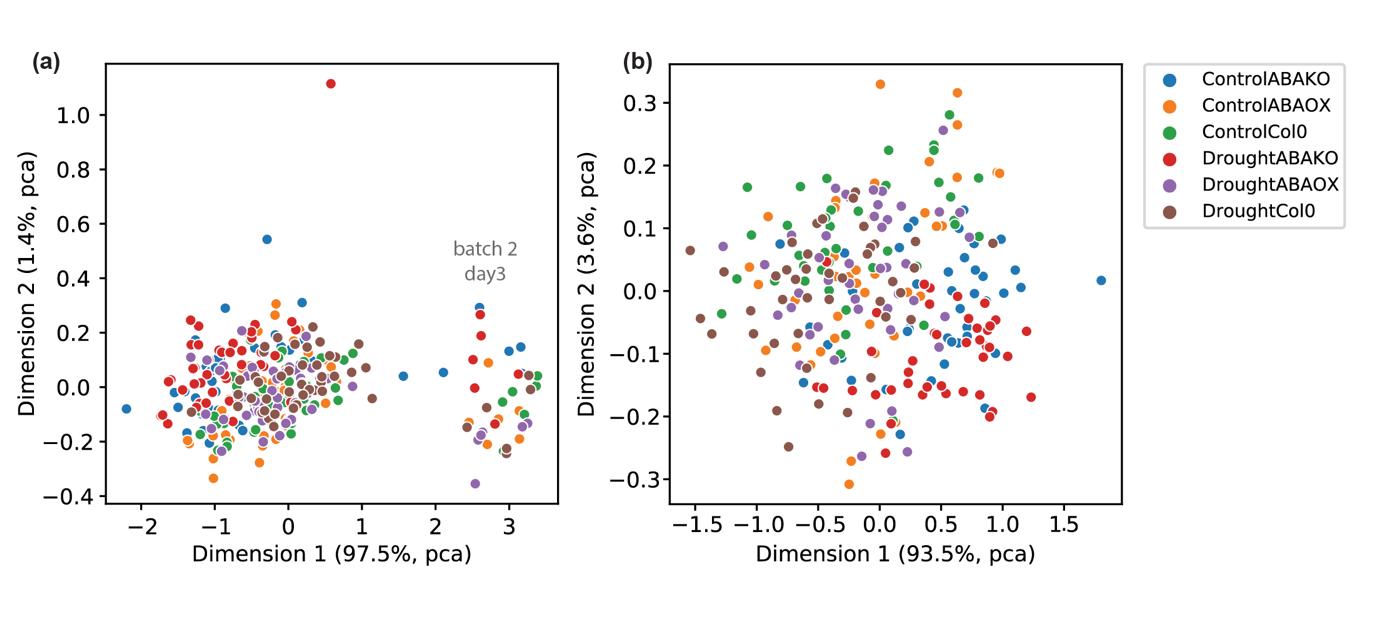
**

**Figure S1. Overview of leaf reflectance data obtained from Arabidopsis Col-0, ABA2-ox and *aba2* plants under control and drought stress.** (**a, b**) Principal Component Analysis of leaf reflectance spectra (average of individual leaves from each condition) from all batches of data collection including outliers (**a**), and excluding outliers (**b**). The batch 2 data at day 3 and other outliers were removed using Isolation Forest method.

**
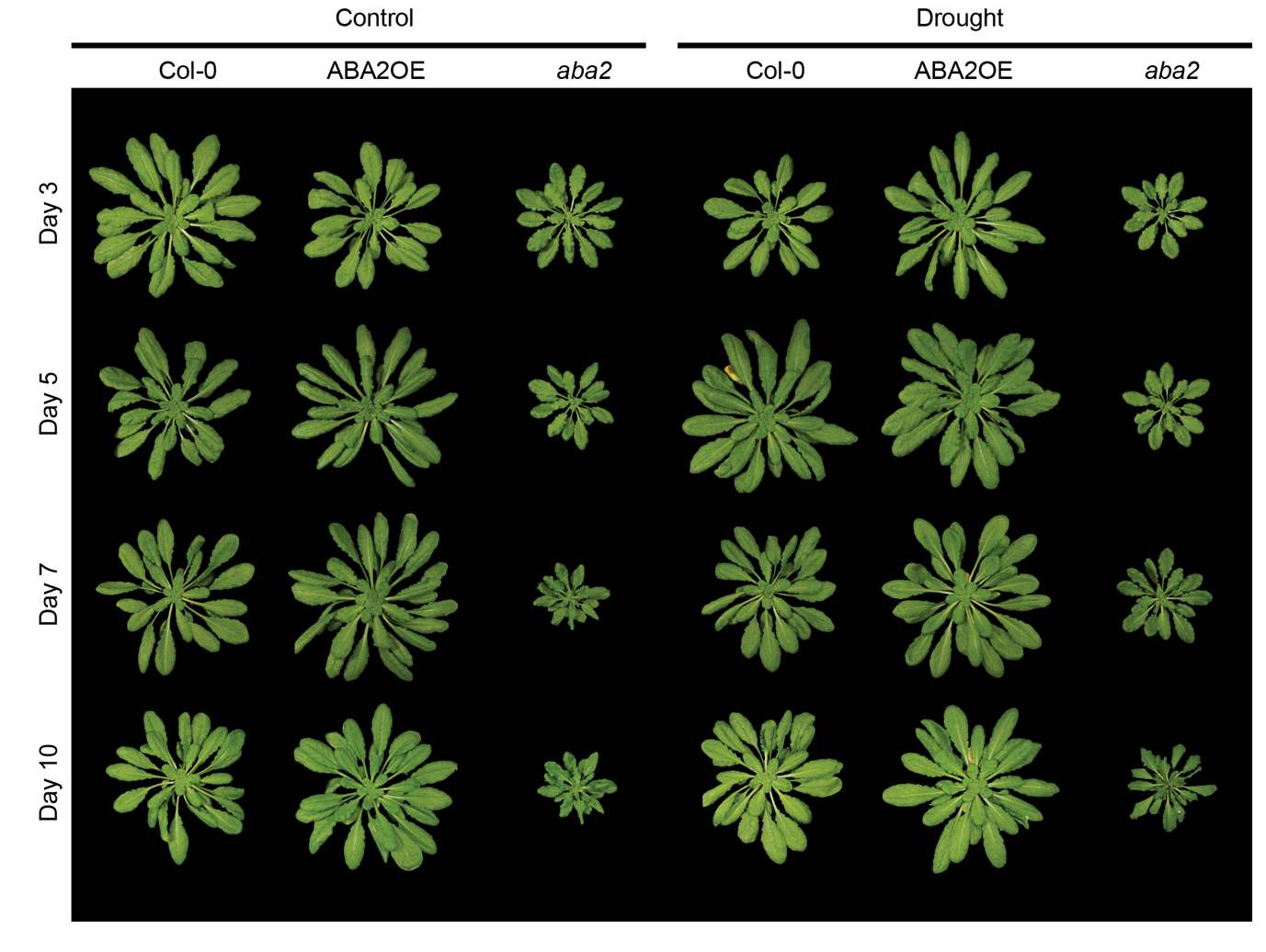
**

**Figure S2. Arabidopsis Col-0, ABA2-ox and *aba2* plant phenotypes.** Representative images of Arabidopsis wildtype Col-0 and transgenic lines ABA2-ox and *aba2* under well-watered and drought conditions for 3, 5, 7, and 10 days (top to bottom). The day 5 photos are represented in Figure 1a.

**
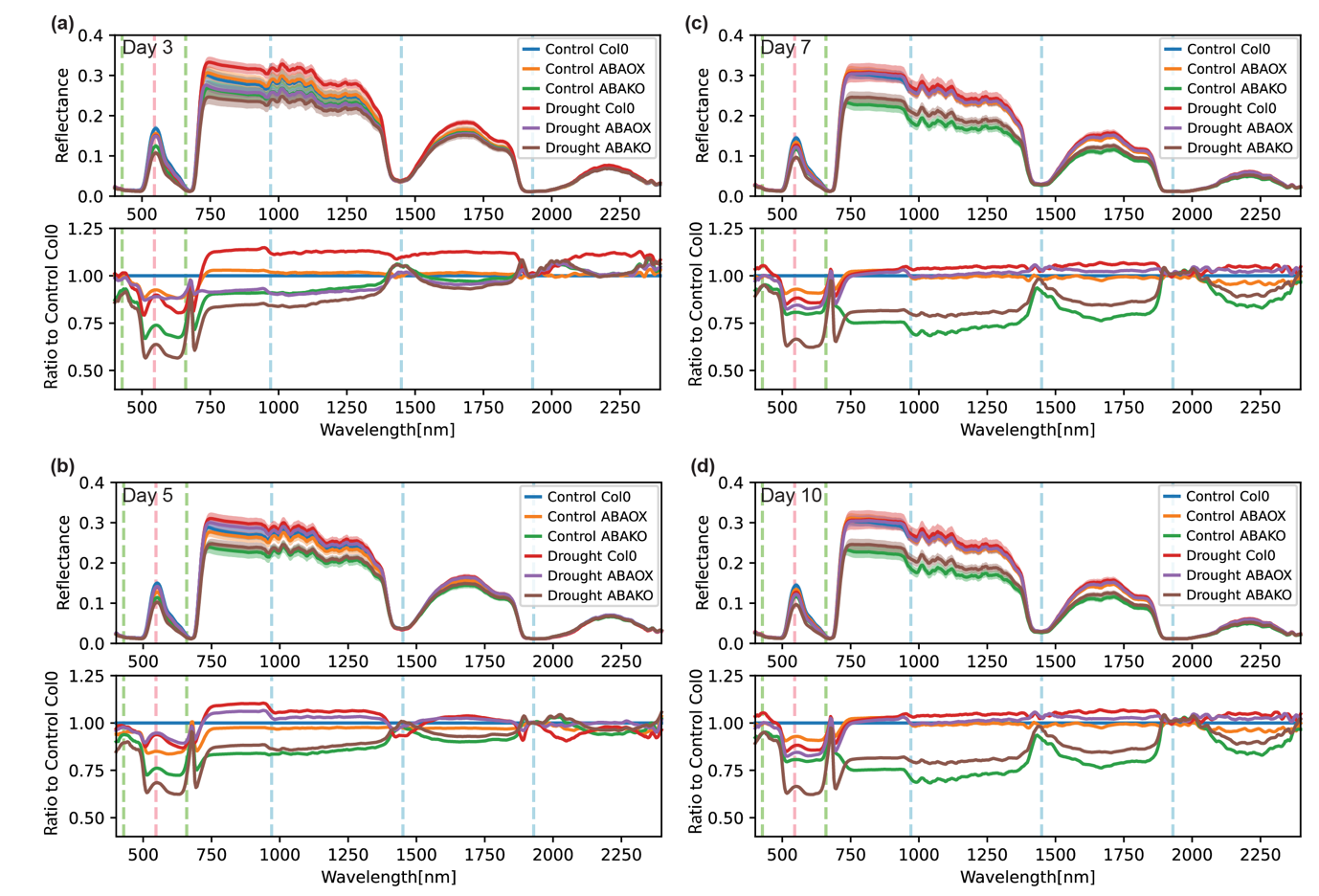
**

**Figure S3. Leaf reflectance spectra from Arabidopsis plants under control and drought stress conditions**. Leaf reflectance spectra from Col-0, *aba2* and ABA2-ox plants under control and drought conditions at day 3 (**a**), day 5 (**b**), day 7 (**c**), and day 10 (**d**). Solid line and transparent band represent the mean value and 95% confidence interval respectively. The bottom panels show the reflectance ratio of the mean values of individual groups to those of Col-0 control. Dashed vertical lines represent absorption spectra for chlorophyll (428, 660 nm, green), anthocyanin (546 nm, red), and water (970, 1450, 1930 nm, blue).

**
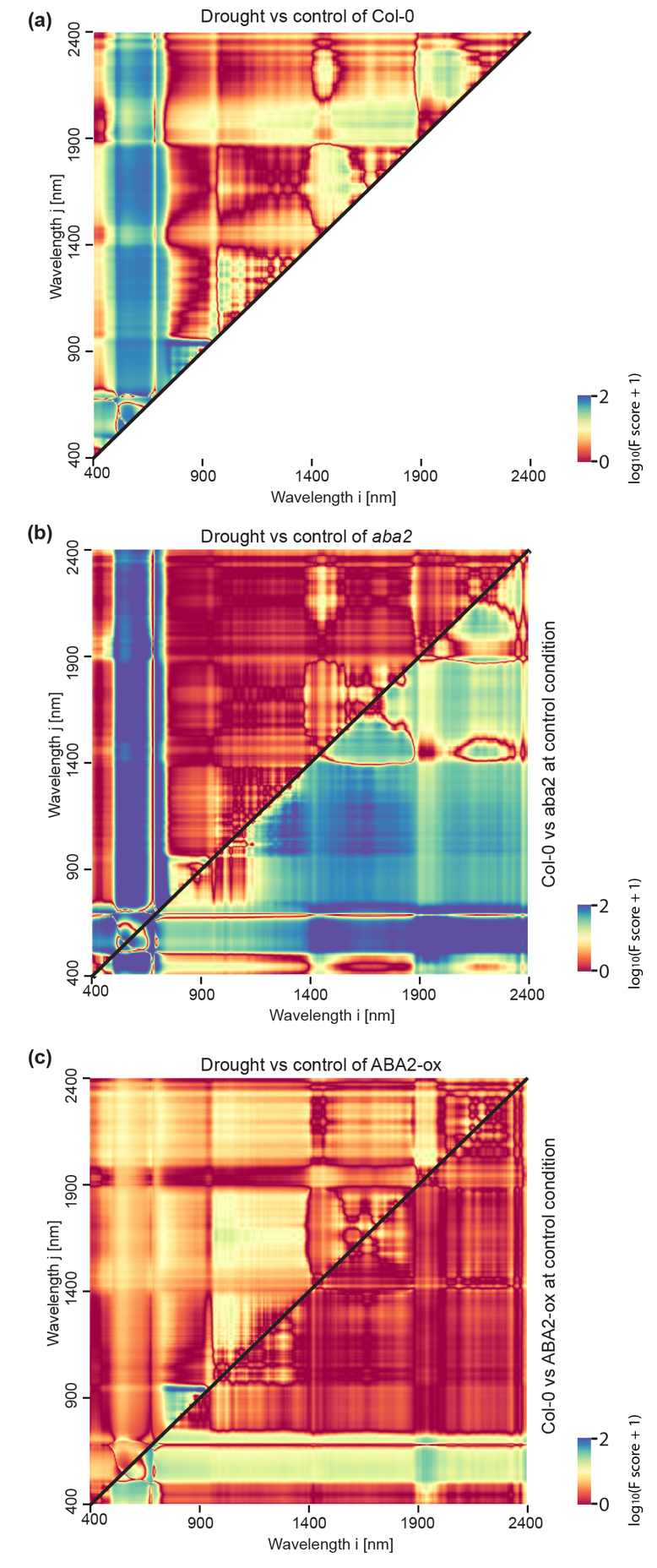
**

**Figure S4. *De novo* screening of drought- and genotype-oriented spectral features**. *De novo* normalized reflectance indices. Heatmap shows ANOVA F values for NRI selection for classification models. (**a**) Drought to control condition in Col-0 (top left triangle) (**b**) Drought to control in *aba2* (top left triangle), and Col-0 vs *aba2* under control conditions (bottom right triangle). (**c**) Drought to control in ABA2-ox (top left triangle), and Col-0 to ABA2-ox at control condition (bottom right triangle).


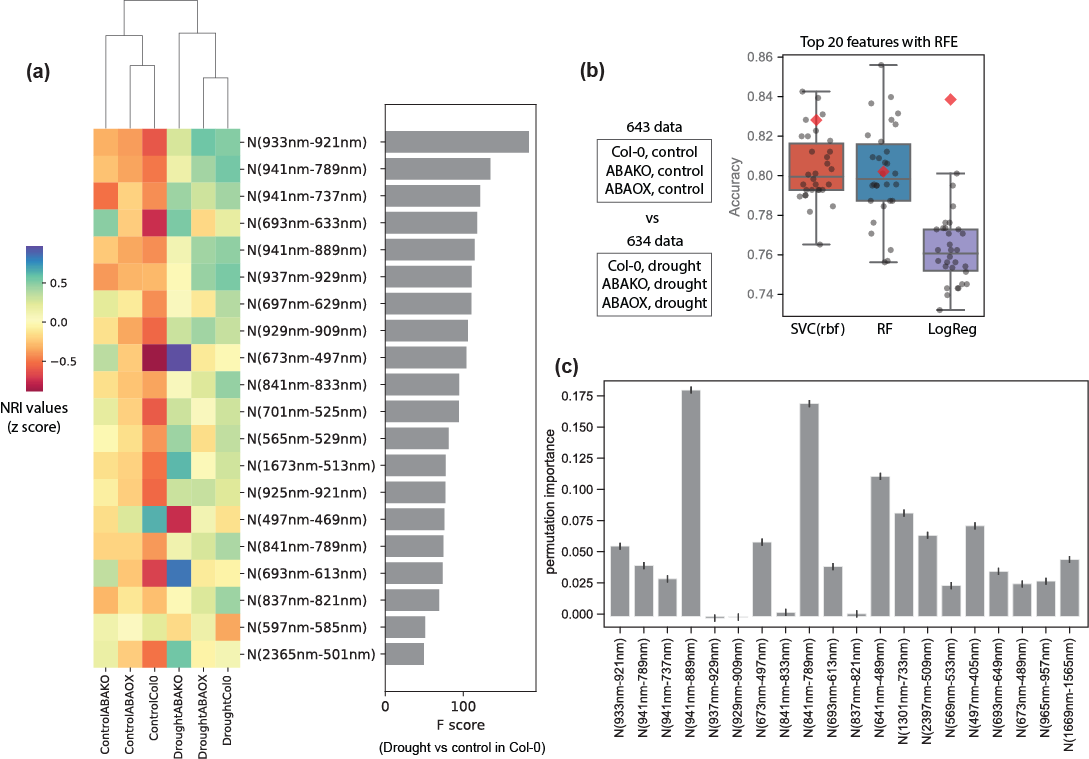


**Figure S5. Selection of normalized reflectance indices for control vs drought comparison.** (**a**) Non-redundant NRIs selected based on the control vs drought comparison in Col-0 using ANOVA F-value scores and PCC. Top 20 NRIs were further selected with the RFE method. Heatmap shows z scored-normalized values of the top 20 NRIs across six tested groups (3 genotypes x 2 conditions). The bar graph on the right shows F scores of the selected features. (**b, c**) Classification models to separate watering and drought groups. Classifiers were built with SVM, RF and LogReg algorithms. Boxplots show classification accuracy with the training set by the Repeats Stratified K-Fold cross validation (n = 30, black dots). Red diamond shows the accuracy with the testing set. Permutation importance of NRIs for LogReg model is shown in (**c**). Bar graph in (**c**) shows the mean ± s.d. derived from 25 permutation tests.


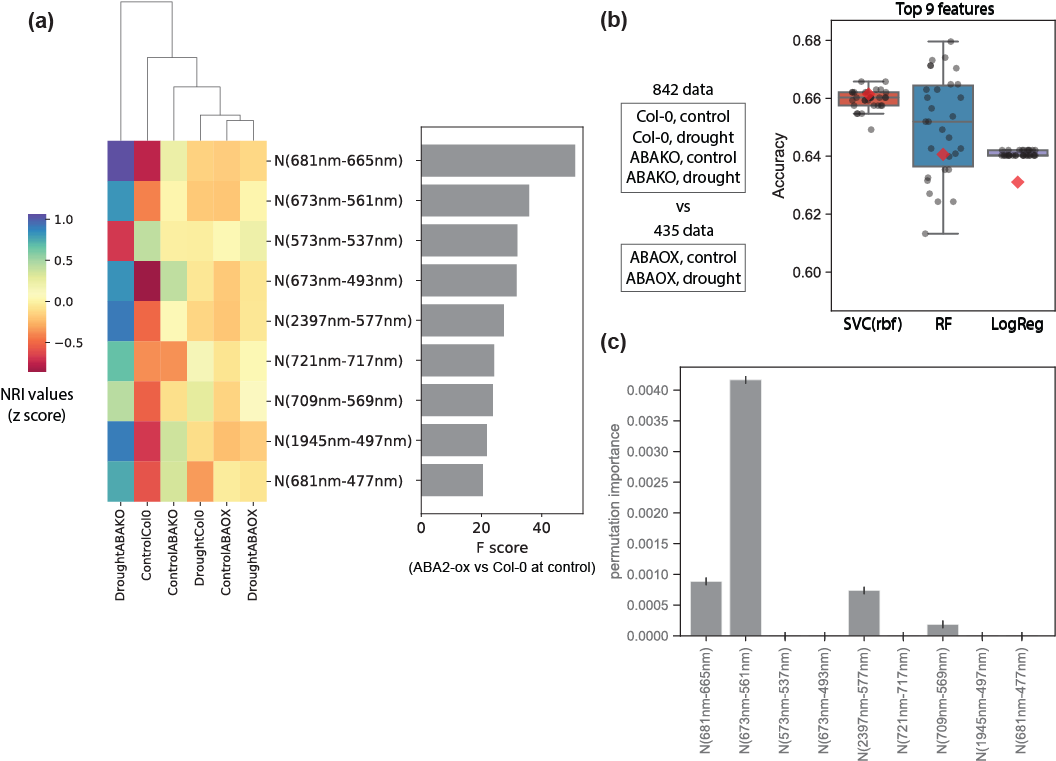


**Figure S6.** **Selection of normalized reflectance indices oriented from *ABA2*-ox.** (**a**) Non-redundant NRIs selected based on the ABA2-ox vs Col-0 comparison using ANOVA F-value scores and PCC. Top 20 NRIs were further selected with the RFE method. Heatmap shows z scored-normalized values of the top 20 NRIs across six tested groups (3 genotypes x 2 conditions). The bar graph on the right shows F scores of the selected features. (**b, c**) Classification models to separate *ABA2*-OX mutant from the other two genotypes. Classifiers were built with SVM, RF and LogReg algorithms. Boxplots show classification accuracy with the training set by the Repeats Stratified K-Fold cross validation (n = 30, black dots). Red diamond shows the accuracy with the testing set. Permutation importance of NRIs for SVM model is shown in (**c**). Bar graph in (**c**) shows the mean ± s.d. derived from 25 permutation tests.


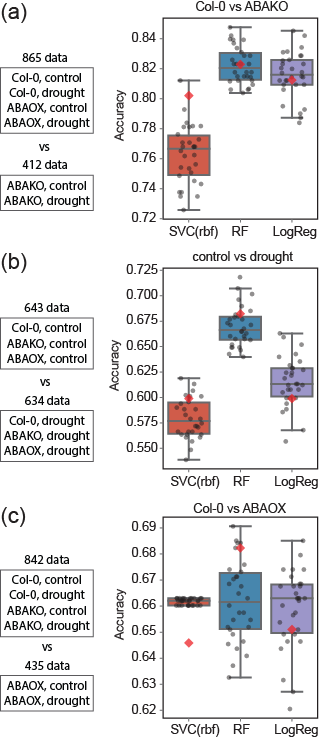


**Figure S7. Classification accuracy using multiple well-established NRIs relating to chlorophyll and water content.** (**a, b, c**) Classification models to separate Col-0 vs *aba2* (ABAKO) (a) and ABA overexpression mutants (**c**), and to separate control vs. drought (**b**), using well-established NRIs relating to chlorophyll and water content. Classifiers were built with SVM, RF and LogReg algorithms. Boxplots show classification accuracy with the training set by the Repeats Stratified K-Fold cross validation (n = 30, black dots). Red diamond shows the accuracy with the testing set.


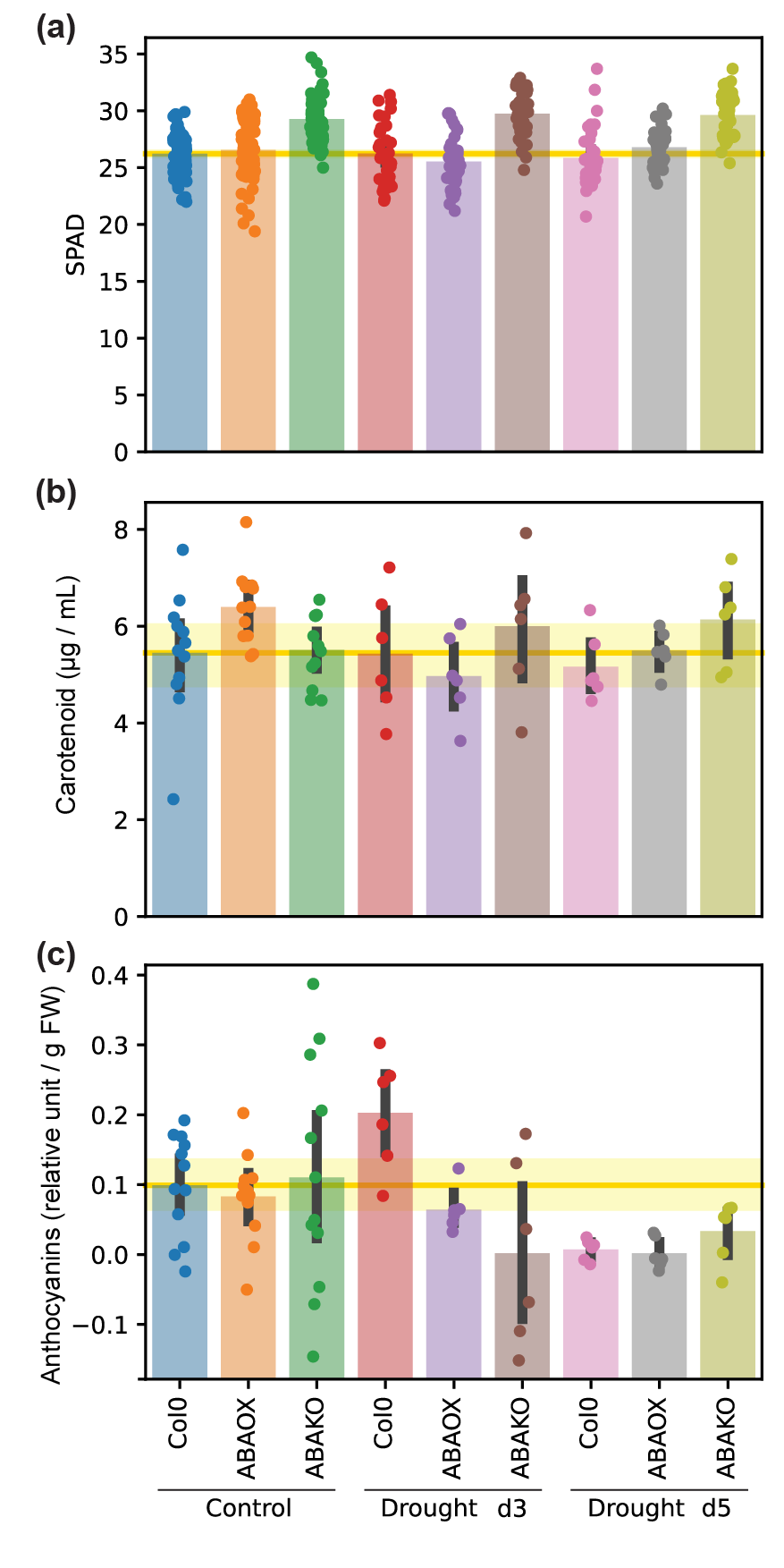


**Figure S8. Quantification of chlorophyll, carotenoid, and anthocyanin contents in Arabidopsis leaves.** The graphs show (**a**) Chlorophyll content, (**b**) Carotenoid content, and (**c**) Anthocyanin content of Arabidopsis plants under control and drought stress conditions. Individual points show raw data, bars show mean, while error bars show standard deviation. Control (watering) condition datapoints were combined for days 3 and 5. The horizontal yellow line shows the mean of Col-0, while the yellow band shows the 95% C.I. of Col-0. A total of n = 6 (chlorophyll, anthocyanins) and n = 9 (leaf water content) biological replicates were collected over 2 (chlorophyll, anthocyanins) or 3 (leaf water content) independent rounds of experiments. The mean of two SPAD readings per leaf are plotted (2 datapoints per biological replicate).


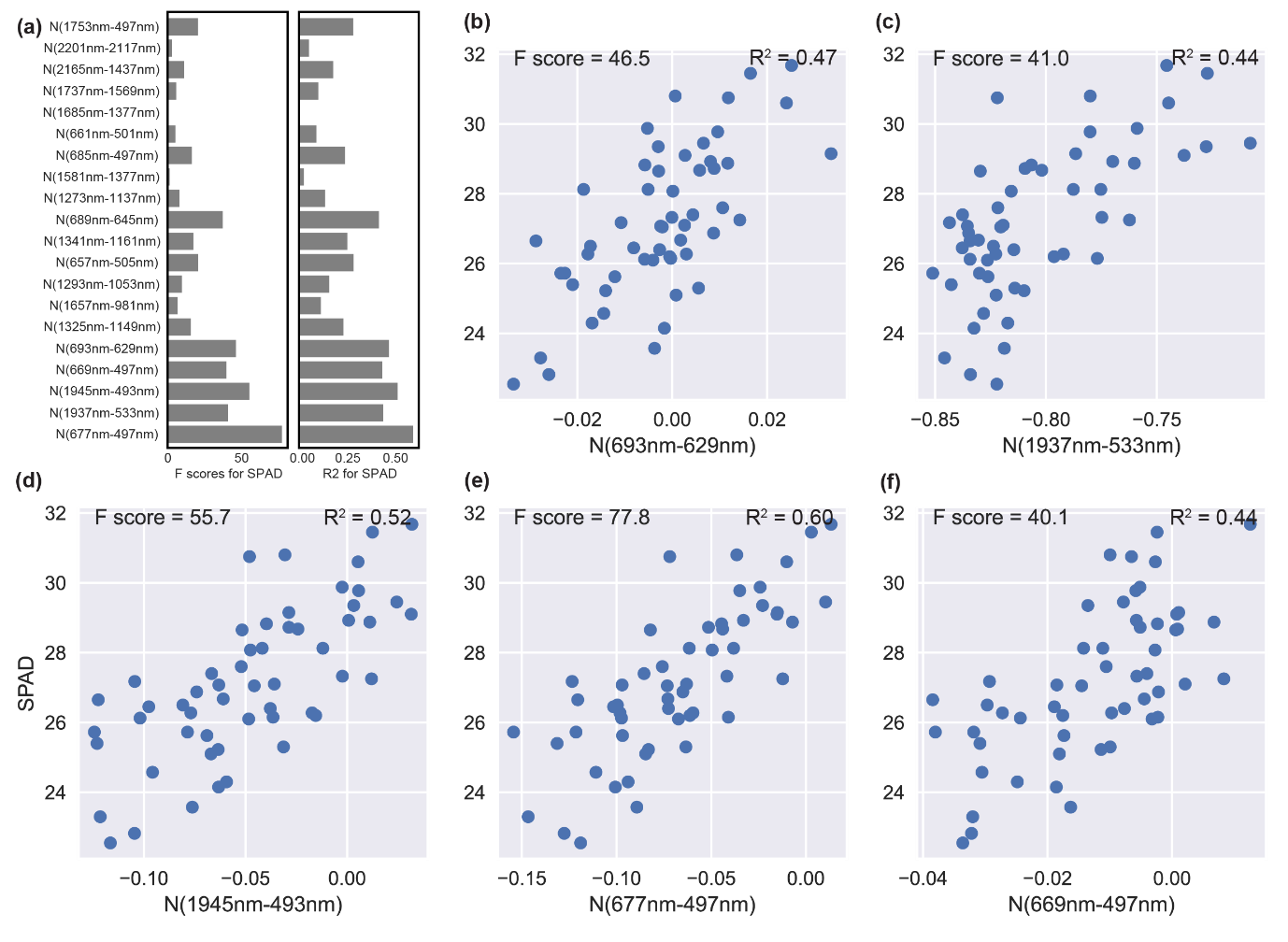


**Figure S9. Correlation of selected *de novo* NRIs with chlorophyll content measurements.** (**a**) ANOVA F-value and R^2^ scores of selected features for regression. The finalized 20 NRIs were selected based on the comparison between *aba2* and Col-0/ABA2-ox with RFE method. (**b-f**) Scatter plots showing chlorophyll content data and the following top five features: N(693 nm – 629 nm) (**b**), N(677 nm – 497 nm) (**c**), N(1945 nm – 493 nm) (**d**), N(1937 nm – 533 nm) (**e**), and N(689 nm – 645 nm) (**f**). The data in (**a**) and (**c**) was reorganised and presented in Figure 3.


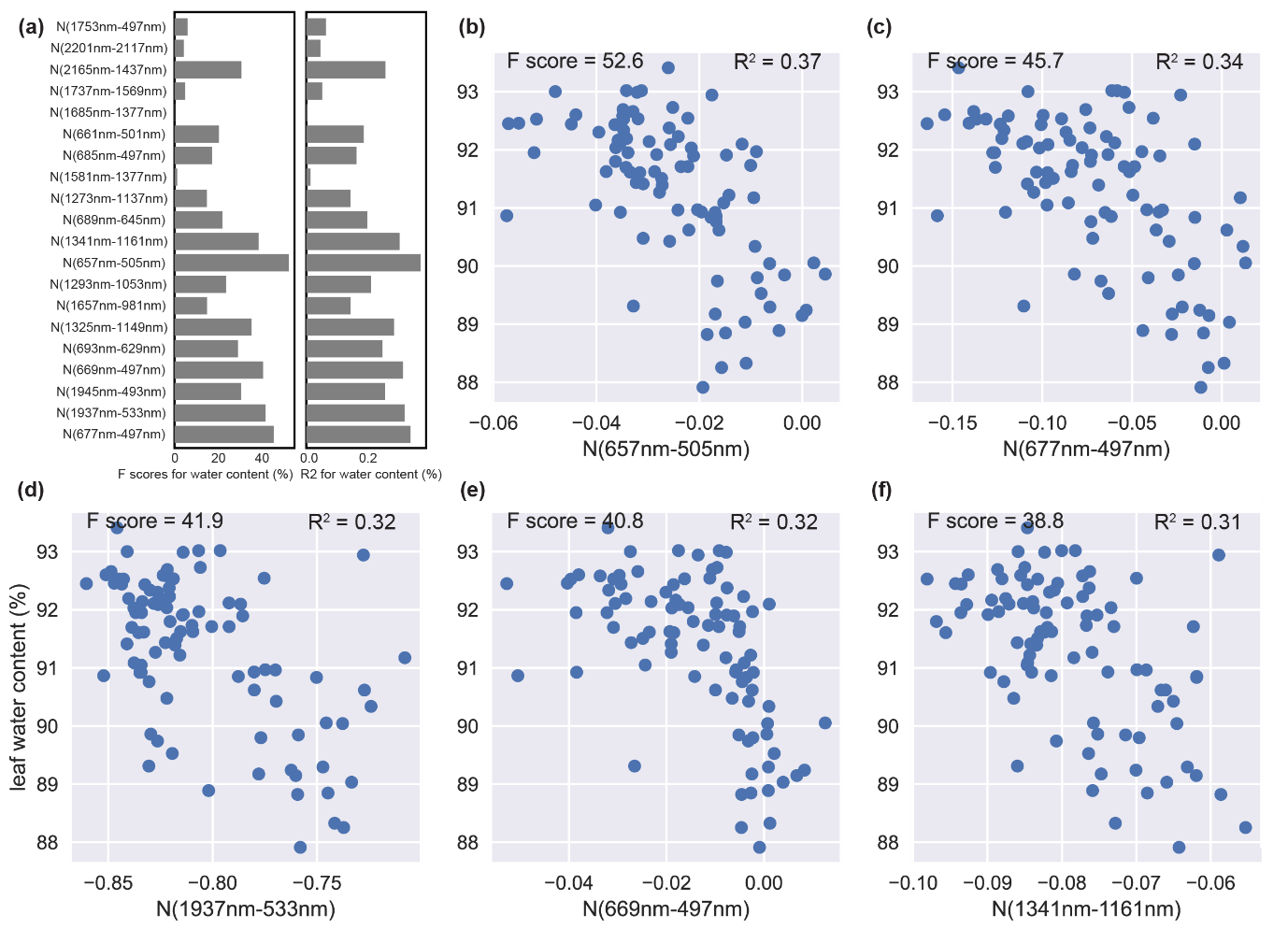


**Figure S10. Correlation of selected *de novo* NRIs with leaf water content measurements.** (**A**) ANOVA F-value and R^2^ scores of selected features for regression. (**b-f**) Scatter plots showing leaf water content data and the following top five features: N(677 nm – 477 nm) (**b**), N(657 nm – 505 nm) (**c**), N(677 nm – 497 nm) (**d**), N(1937 nm – 533 nm) (**e**), and N(669 nm – 497 nm) (**f**). The data in (**a**) and (**c**) was reorganised and presented in Figure 3.


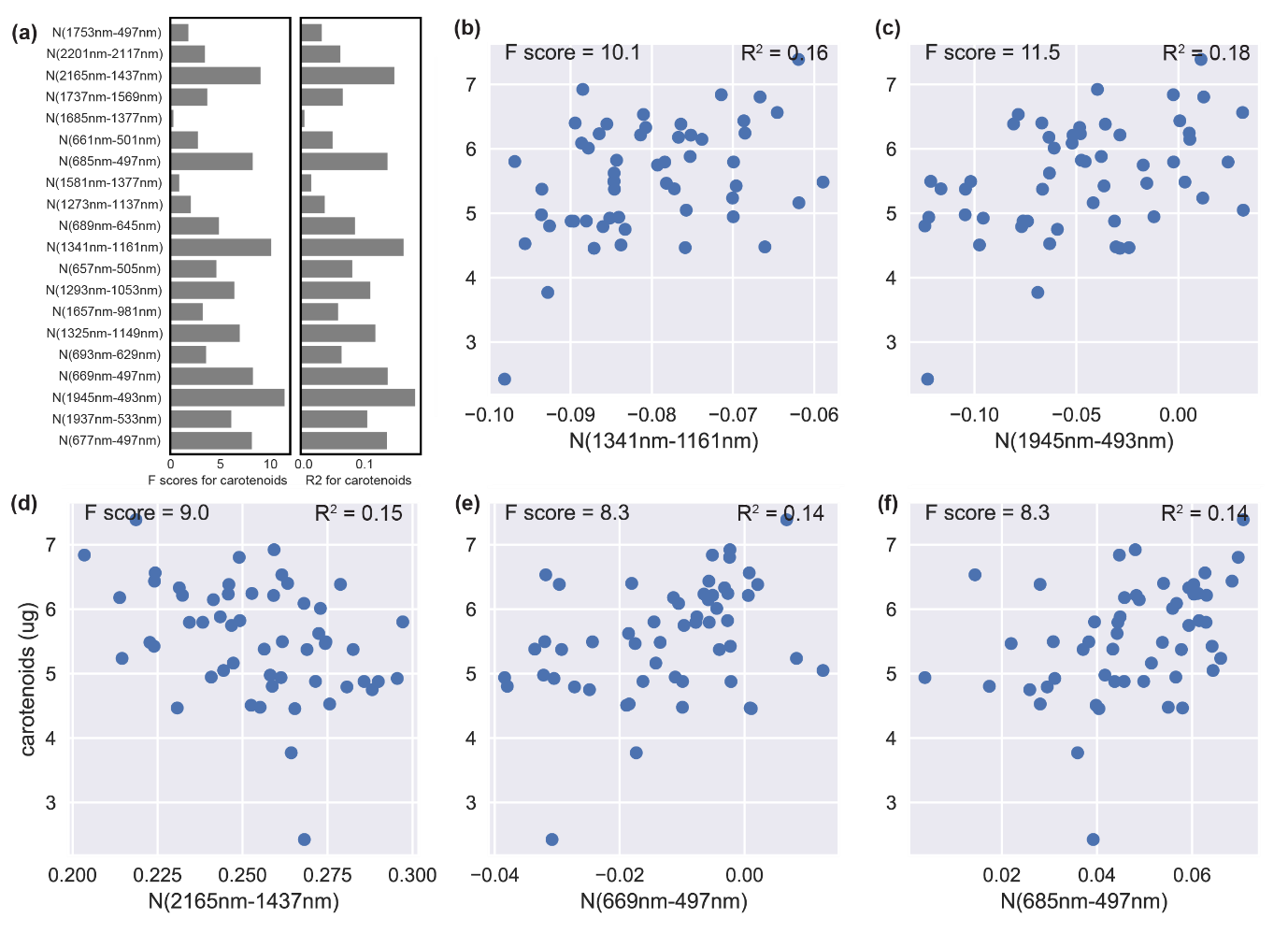


**Figure S11. Correlation of selected *de novo* features with carotenoid content measurements.** (**a**) ANOVA F-value and R^2^ scores of selected features for regression. (**b-f**) Scatter plots showing carotenoid content data and the following top five features: N(1945 nm – 493 nm) (**b**), N(1341 nm – 1161 nm) (**c**), N(669 nm – 497 nm) (**d**), N(685 nm – 497 nm) (**e**), and N(677 nm – 497 nm) (**f**). The data in (**a**) was reorganised and presented in Figure 3.


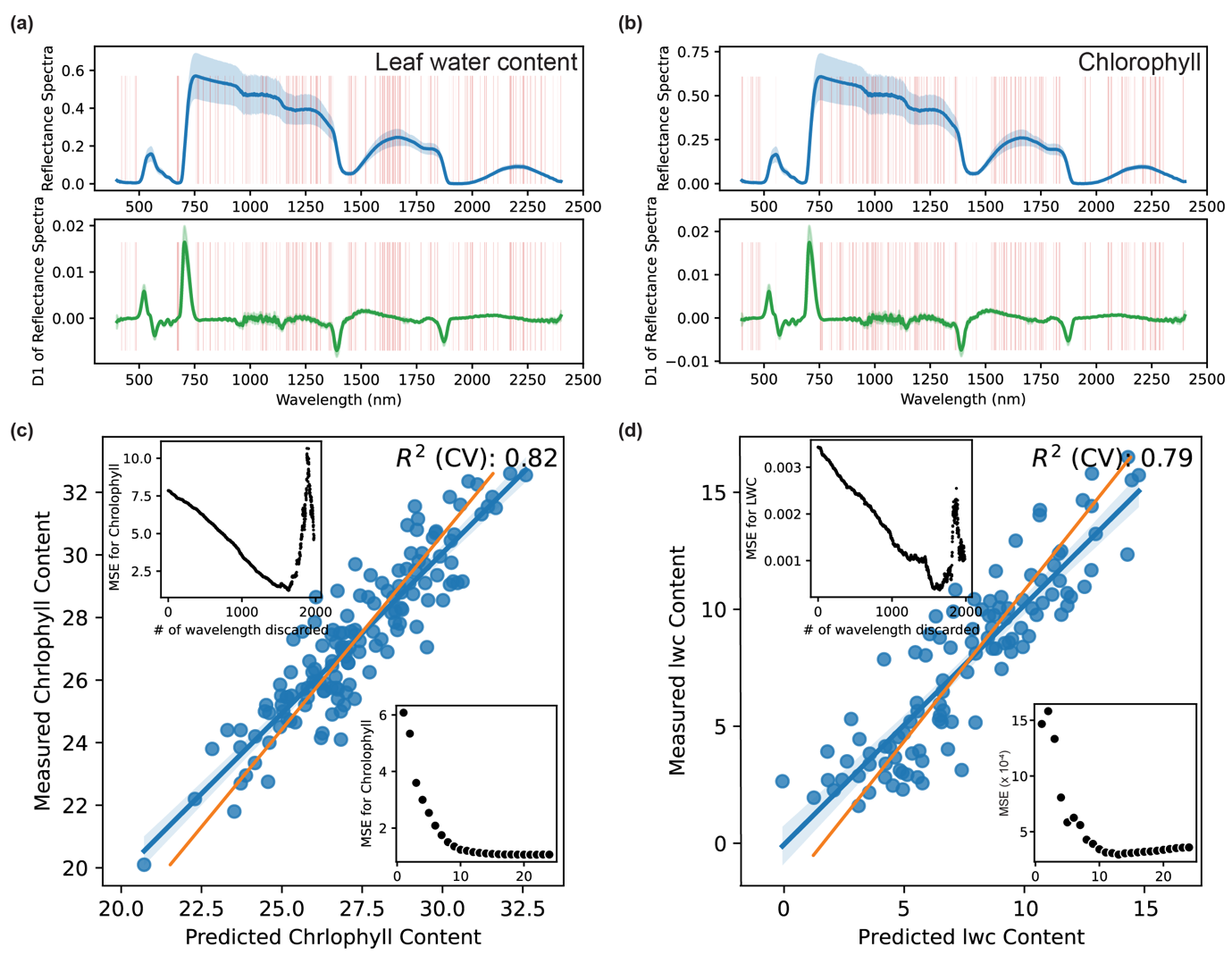


**Figure S12. PLS regression models for leaf water and chlorophyll content.** (**a, b**) Original (top) and first derivative (bottom) leaf reflectance spectra are plotted. Red lines represent wavelengths that were excluded during the building of the partial least squares regression models for leaf water content (**a**) and chlorophyll content (**b**). Solid line and transparent band represent the mean value and 95% confidence interval respectively. (**c, d**) Partial least squares regression models built for predicting chlorophyll content (**c**) and leaf water content (**d**). Top left plot represents the changes of mean square error (MSE) against the number of wavelengths discarded in building the regression model. Bottom right graph plots the number of PLS components used against the mean square error. Blue line represents the calibration result, while orange represents the cross-validation result. The R^2 value represents the cross-validation result.


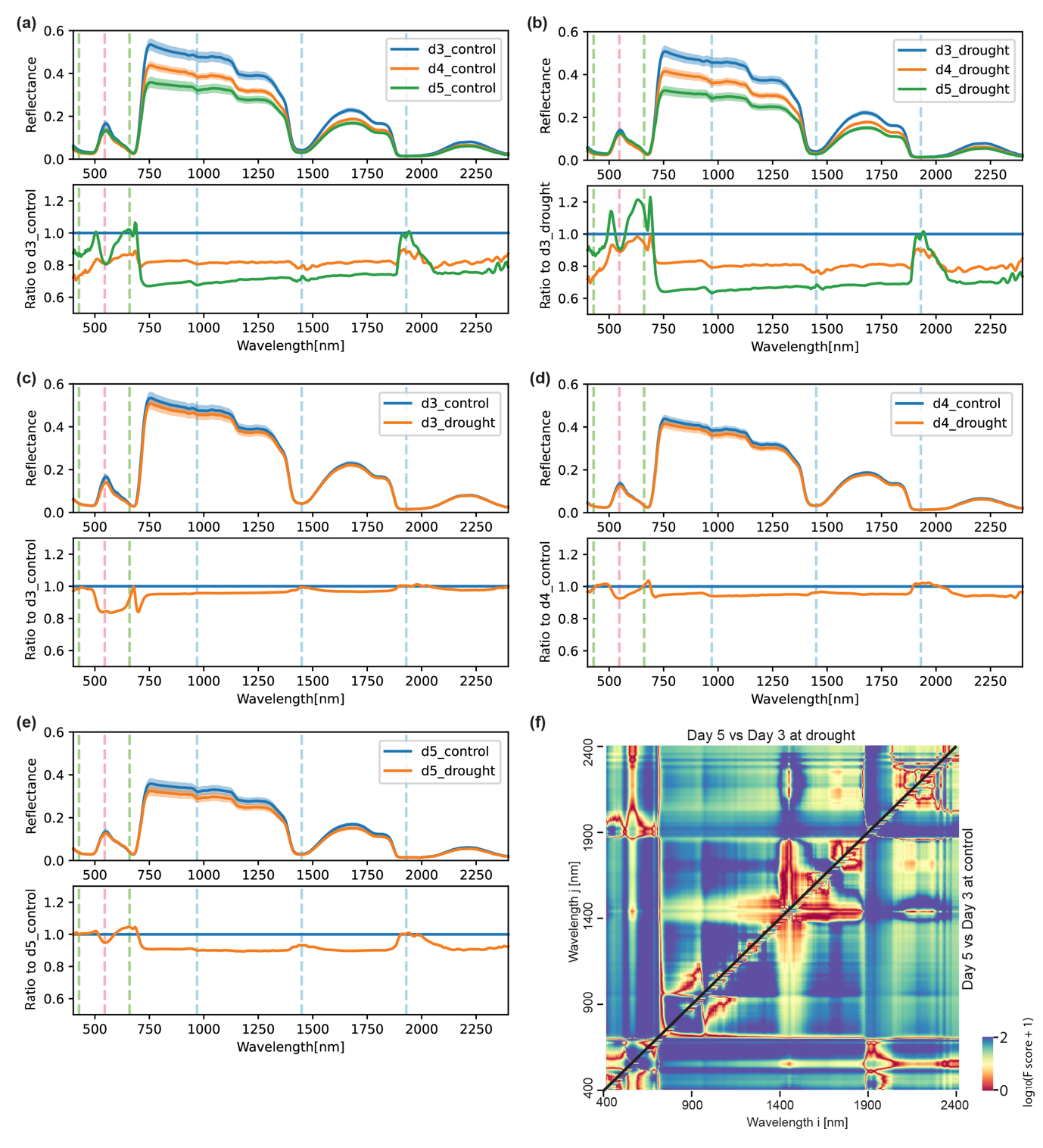


**Figure S13. Leaf reflectance data for lettuce.** (**a**) Comparison between control samples from days 3, 4, and 5 of treatment. (**b**) Leaf spectral comparison between days 3, 4, and 5 of drought treatment. (**c-e**) Comparison between control and drought conditions at day 3 (**c**), day 4 (**d**) and day 5 (**e**). Reflectance ratios are presented on the bottom half. Solid line and transparent band represent the mean value and 95% confidence interval respectively. (**f**) *De novo* NRIs related to leaf age. Heatmap shows ANOVA F values: day 5 to day 3 data under drought (top left triangle) and control conditions (bottom right triangle).

**
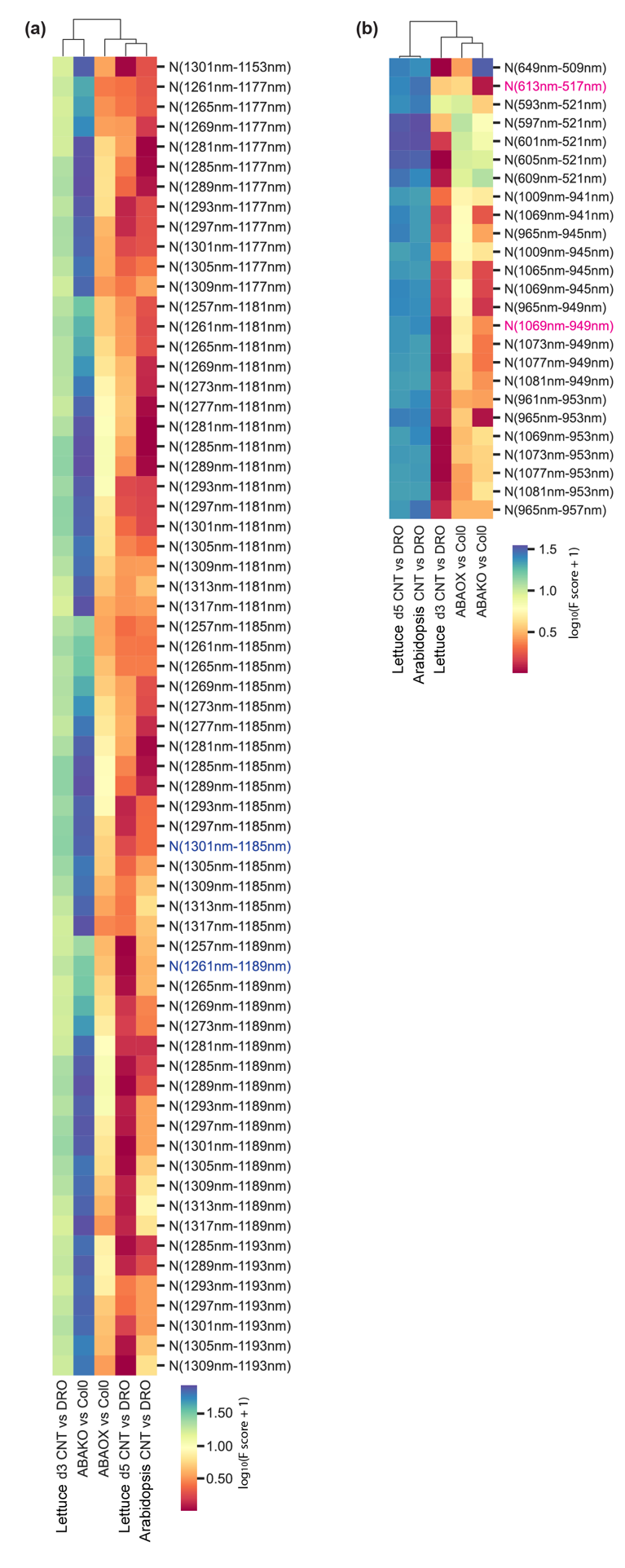
**

**Figure S14. Similarity of selected NRIs in lettuce to those in Arabidopsis.** NRIs related to watering conditions in lettuce at day 3 (**a**) and at day 5 (**b**) without excluding non-redundant NRIs. Heatmaps show the F-value scores of the control vs drought comparisons in Arabidopsis and lettuce, and the Col-0 and mutant comparisons in Arabidopsis. NRIs with blue and pink texts are also presented in Figure 4d.


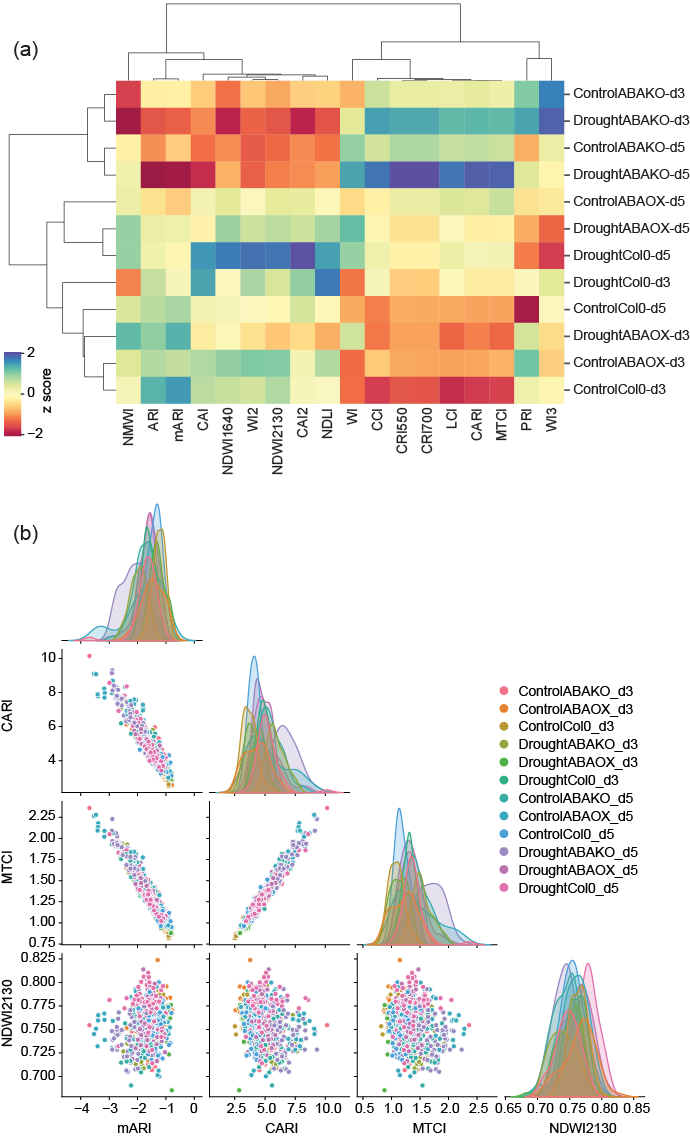


**Figure S15. Conventional agricultural indices associated with plant drought stress.** (**a**) Heatmap showing z score-normalized indices across 12 groups (3 genotypes x 2 conditions x 2 treatment lengths). Conventional agricultural indices were calculated as described in Materials and Methods. (**b**) Pair plots showing cross correlation among agricultural indices related to anthocyanin (mARI), carotenoid (CARI), chlorophyll (MTCI) and water (NDWI2130) contents.

**References**

1. A. A. Gitelson, M. N. Merzlyak, O. B. Chivkunova, Optical Properties and Nondestructive Estimation of Anthocyanin Content in Plant Leaves¶. *Photochemistry and Photobiology* **74**, 38-38 (2001).

2. A. A. Gitelson, G. P. Keydan, M. N. Merzlyak, Three‐band model for noninvasive estimation of chlorophyll, carotenoids, and anthocyanin contents in higher plant leaves. *Geophysical Research Letters* **33**, 2006GL026457-022006GL026457 (2006).

3. A. A. Gitelson, Y. Zur, O. B. Chivkunova, M. N. Merzlyak, Assessing carotenoid content in plant leaves with reflectance spectroscopy. *Photochem Photobiol* **75**, 272-281 (2002).

4. X. Zhou *et al.*, Assessment of leaf carotenoids content with a new carotenoid index: Development and validation on experimental and model data. *International Journal of Applied Earth Observation and Geoinformation* **57**, 24-35 (2017).

5. G. G. Drolet *et al.*, A MODIS-derived photochemical reflectance index to detect inter-annual variations in the photosynthetic light-use efficiency of a boreal deciduous forest. *Remote Sensing of Environment* **98**, 212-224 (2005).

6. J. A. Gamon *et al.*, A remotely sensed pigment index reveals photosynthetic phenology in evergreen conifers. *Proc Natl Acad Sci U S A* **113**, 13087-13092 (2016).

7. R. Pu, P. Gong, Q. Yu, Comparative Analysis of EO-1 ALI and Hyperion, and Landsat ETM+ Data for Mapping Forest Crown Closure and Leaf Area Index. *Sensors* **8**, 3744-3766 (2008).

8. J. Dash, P. J. Curran, The MERIS terrestrial chlorophyll index. *International Journal of Remote Sensing* **25**, 5403-5413 (2004).

9. C. Wu *et al.*, Remote estimation of gross primary production in wheat using chlorophyll-related vegetation indices. *Agricultural and Forest Meteorology* **149**, 1015-1021 (2009).

10. J. Penuelas, J. Pinol, R. Ogaya, I. Filella, Estimation of plant water concentration by the reflectance Water Index WI (R900/R970). *International Journal of Remote Sensing* **18**, 2869-2875 (1997).

11. H.-D. Seelig *et al.*, Plant water parameters and the remote sensing R1300/R1450 leaf water index: controlled condition dynamics during the development of water deficit stress. *Irrigation Science* **27**, 357-365 (2009).

12. R. R.-P. José, R. David, C. Eli, U. Susan, R. S. David, Evaluation of Hyperspectral Reflectance Indexes to Detect Grapevine Water Status in Vineyards. *American Journal of Enology and Viticulture* **58**, 302 (2007).

13. D. Chen, J. Huang, T. J. Jackson, Vegetation water content estimation for corn and soybeans using spectral indices derived from MODIS near- and short-wave infrared bands. *Remote Sensing of Environment* **98**, 225-236 (2005).

14. P. L. Nagler, Y. Inoue, E. P. Glenn, A. L. Russ, C. S. T. Daughtry, Cellulose absorption index (CAI) to quantify mixed soil–plant litter scenes. *Remote Sensing of Environment* **87**, 310-325 (2003).

15. P. L. Nagler, C. S. T. Daughtry, S. N. Goward, Plant Litter and Soil Reflectance. *Remote Sensing of Environment* **71**, 207-215 (2000).

16. L. Serrano, J. Peñuelas, S. L. Ustin, Remote sensing of nitrogen and lignin in Mediterranean vegetation from AVIRIS data: Decomposing biochemical from structural signals. *Remote Sensing of Environment* **81**, 355-364 (2002).
